# Assessment of Heterozygosity and Genome-Wide Analysis of Heterozygosity Regions in Two Duroc Pig Populations

**DOI:** 10.3389/fgene.2021.812456

**Published:** 2022-01-27

**Authors:** Donglin Ruan, Jie Yang, Zhanwei Zhuang, Rongrong Ding, Jinyan Huang, Jianping Quan, Ting Gu, Linjun Hong, Enqin Zheng, Zicong Li, Gengyuan Cai, Xiaopeng Wang, Zhenfang Wu

**Affiliations:** ^1^ College of Animal Science and National Engineering Research Center for Breeding Swine Industry, South China Agricultural University, Guangzhou, China; ^2^ Guangdong Provincial Laboratory of Lingnan Modern Agricultural Science and Technology, Guangzhou, China; ^3^ Guangdong Wens Breeding Swine Technology Co., Ltd., Yunfu, China

**Keywords:** Duroc pigs, single nucleotide polymorphism, runs of heterozygosity, economic traits, association analysis

## Abstract

Heterozygosity can effectively reflect the diverse models of population structure and demographic history. However, the genomic distribution of heterozygotes and the correlation between regions of heterozygosity (runs of heterozygosity, ROHet) and phenotypes are largely understudied in livestock. The objective of this study was to identify ROHet in the Duroc pig genome, and investigate the relationships between ROHet and eight important economic traits. Here, we genotyped 3,770 American Duroc (S21) and 2,096 Canadian Duroc (S22) pigs using 50 K single nucleotide polymorphism array to analyze heterozygosity. A total of 145,010 and 84,396 ROHets were characterized for S21 and S22 populations, respectively. ROHet segments were mostly enriched in 1–2 Mb length classification (75.48% in S21 and 72.25% in S22). The average genome length covered by ROHet was 66.53 ± 12.20 Mb in S21 and 73.32 ± 13.77 Mb in S22 pigs. Additionally, we detected 20 and 13 ROHet islands in S21 and S22 pigs. Genes in these genomic regions were mainly involved in the biological processes of immunity and reproduction. Finally, the genome-wide ROHet-phenotypes association analysis revealed that 130 ROHets of S21 and 84 ROHets of S22 were significantly associated with eight economic traits. Among the candidate genes in the significant ROHet regions, 16 genes related to growth, metabolism, and meat quality were considered as candidate genes for important economic traits of pigs. This work preliminarily explores the effect of heterozygosity-rich regions in the pig genome on production performance and provides new insights for subsequent research on pig genetic improvement.

## Introduction

Pork is one of the most consumed meats in the world and the main source of animal protein for humans. Furthermore, Duroc × (Landrace × Yorkshire) (DLY) commercial pigs dominate the pork market in China (Wu et al., 2021). Among them, Duroc pigs provide half of the genetic contribution in DLY pigs. Due to the characteristics of fast growth rate, good meat quality, and high feed conversion efficiency, Duroc pigs are widely used in commercial pig breeding worldwide (Lin et al., 2019). Combining the Duroc pig genome with genetic improvement of important traits has always been the focus of research for breeders.

With the development of DNA sequencing technology and the reduction in the cost of sequencing and genotyping, whole-genome molecular markers provide powerful tools for studying animal genomes. Tens of thousands of single nucleotide polymorphism (SNP) markers covering the whole pig genome have facilitated the use of molecular breeding strategies such as genomic selection (GS) (Uemoto et al., 2017) and genome-wide association study (GWAS) (Zhang et al., 2021), which has rapidly improved the breeding process of Duroc pigs. In addition, genomic data was utilized to explore selection signatures and reveal potential genetic mechanisms underlying the domestication and evolution of Duroc pigs (Kim et al., 2015; Zhang et al., 2020). For instance, runs of homozygosity (ROH) were widely applied to identify candidate regions and genes in the pig genome that were subject to selection and associated with important traits (carcass and reproduction traits) (Hong et al., 2021), because positive selection reduces genetic diversity and increases homozygosity (Fang et al., 2021). These methods have contributed to elucidating the genetic architecture of Duroc pigs, primarily focusing on haplotypes and homozygosity. However, few studies have been conducted on heterozygosity-rich regions of the Duroc pig genome.

The estimating of heterozygosity provides a valuable but often neglected resource for the study of genetic variability and evolutionary history (Samuels et al., 2016). Recent studies have highlighted the role of runs of heterozygosity (ROHet) analysis in the research of livestock and poultry genome. ROHet were first reported in 2015 and defined as a segment of the genome that is continuous with the heterozygous genotype (Williams et al., 2016). Compared to heterozygosity (*H*) in population genetics, ROHet were a better indicator of the distribution of heterozygotes in the genome and could be used to study selection, introgression, and hypervariable segments (Marras et al., 2018). Besides, ROHet avoid the deleterious effects of harmful homozygous genotype aggregation on specific traits and improve fitness. For example, a significant ROHet region detected in the horse genome contains *TRIM37*, *PPM1E,* and *CA10* genes that probably are associated with gait trait (Bizarria Dos Santos et al., 2021). In addition, the *GABRB1*, *TARSL2*, *TM2D3*, *PCSK6,* and *SNRPA1* genes in the high frequency ROHets of the Maremmana cattle genome were associated with fitness and reproductive traits (Biscarini et al., 2020). However, there were still relatively few reports of ROHet in commercial livestock breeds, and no studies have been conducted to detect ROHet in pigs. Moreover, previous studies mainly used the distribution of ROHet and the function of genes within ROHet to evaluate the effect of ROHet on population characteristics, and there is a lack of investigating the effect of ROHet on phenotypes. Therefore, the aim of this study was to reveal the distribution of heterozygotes within the genome by detecting ROHet in two Duroc populations. Then, this was integrated with the association analysis of ROHet with eight important traits, including 100 kg average daily gain (ADG), 100 kg backfat thickness (BF), body length (BL), birth weight (BW), loin muscle area (LMA), loin muscle depth (LMD), lean meat percentage (LMP), and total teat number (TN), to uncover the relationship between heterozygosity-rich regions and production performances of Duroc pigs.

## Materials and Methods

### Animals and Phenotypes

In the present study, all experimental animals were raised in three core farms of Wens Foodstuff Group CO, Ltd. (Guangdong, China) with the same feeding standards, including 3,770 American Duroc (S21) pigs and 2,096 Canadian Duroc (S22) pigs. The S21 pigs were born between 2013 and 2017 (2,280 males and 1,490 females), and the S22 pigs were born between 2015 and 2017 (1,017 males and 1,079 females). In addition, the phenotypes of ADG, BF, BL, BW, LMA, LMD, LMP, and TN were collected for both populations (Supplementary Table S1).

### Genotyping and Data Editing

Ear tissue was collected from 5,866 Duroc pigs and genomic DNA was extracted using the standard phenol/chloroform method. All DNA samples were genotyped using GeneSeek porcine 50 K SNP chip from Illumina (Neogen, Lincoln, NE, United States), of which 50,649 SNPs were mapped to *Sus scrofa* 11.1. Genotyping data were pruned by PLINK v1.9 (Chang et al., 2015) using the following criteria of exclusion: (1) call rate <90%; (2) minor allele frequency <5%; (3) Hardy-Weinberg test *p*-value < 10^–6^; and (4) SNPs located on sex chromosomes and on unmapped regions. Missing genotypes were imputed by Beagle v5.0 (Browning et al., 2018). After quality control and imputation, a final data set of 39,416 SNPs remained for all pigs for the subsequent analyses.

### Runs of Heterozygosity Detection

In this study, runs of heterozygosity were detected using the R detectRUNS package v0.9.6 (Biscarini et al., 2018) with consecutive strategy. The following parameters were applied to detect ROHet in S21 and S22 pigs respectively: (1) the minimum length of the ROHet was 1Mb; (2) the minimum number of SNPs in a ROHet was 15; (3) max gap between consecutive heterozygous SNP was 10^6^ bp; (4) the maximum of one opposite genotype SNP and no missing SNPs in the run. To better understand the distribution of heterozygous region in the genomes of two Duroc populations, ROHets were classified into three categories: 1-2 Mb, 2–4 Mb, and >4 Mb. The number of ROHet and chromosome distribution were counted. In order to clearly compare the enrichment of ROHet across individuals, we presented the fraction of genome covered by ROHet ( $f_{ROHet}$ ), which is analogous to *F*_ROH_ (Dumitrescu et al., 2005) and computed by:

$$f_{ROHet}=\frac{L_{ROHet}}{L_{gen}},$$

where $L_{ROHet}$ was the total length of ROHet in the genome for each Duroc individual and $L_{gen}$ was total length of the autosomes (2.45 Gb in this study).

Furthermore, we selected SNPs in the top 1% of occurrence to identify highly heterozygous regions (ROHet island), which translated to ROHet frequency of 0.37 and 0.40 for the S21 and S22 populations as thresholds, respectively.

### Genome-wide ROHet Analysis

According to the model described in a previous study (Cesarani et al., 2021), we investigated the association between unique heterozygous regions (presence or absence) and eight economic traits of Duroc pigs via the “-lm” option in GEMMA software v0.98.3 (Zhou and Stephens, 2012). The linear model is as follows:

$$y_{ijklm}=farm_{j}+birthyear_{k}+sex_{l}+ROHet_{m}+e_{ijklm},$$

where

(1) $y_{ijklm}$ is the phenotype of the i ‐ th individual, including ADG, BF, BL, BW, LMA, LMD, LMP, and TN;
(2) $farm_{j}$ is the fixed effect of the j ‐ th farm (one level for S21 population and two levels for S22 population);
(3) $birthyear_{k}$ is the fixed effect of the k ‐ th  birth year, which five levels for S21 population (2013–2017) and three levels for S22 population (2015–2017);
(4) $sex_{l}$ is the fixed effect of the gender with two levels (male and female);
(5) $ROHet_{m}$ is the fixed effect with two levels (presence as one and absence as zero) for each population;
(6) $e_{ijklm}$ is the random residual.

ROHets shared by at least 20 animals in each population would be analyzed (Cesarani et al., 2021), where 842 effective ROHets for the S21 population and 503 effective ROHets for the S22 population. Considering the low number of effective ROHet and Bonferroni correction is a relatively strict criterion for multiple testing, we used false discovery rate (FDR) to determine the threshold *p*-values of ROHet association analysis (Zhuang et al., 2020). The threshold *p*-values were computed as $P=FDR\times \frac{N}{M}$ , where FDR was set as 0.01, N was the number of ROHet with *p*-value < 0.01, and M was the total number of effective ROHet. In order to further understand the genetic architecture between traits, we utilized Genome-wide Complex Trait Analysis (GCTA) (Lee et al., 2012) software to estimate the genetic correlation among eight economic traits in two Duroc populations, respectively.

### Gene Annotation and Functional Enrichment Analyses

The functional genes in ROHet islands and heterozygous regions significantly associated with traits were annotated by the Ensembl database (*Sus scrofa* 11.1, https://www.ensembl.org/biomart). Then, genes located on ROHet island were subjected to Gene Ontology (GO) and Kyoto Encyclopedia of Genes and Genomes (KEGG) enrichment analyses using the KOBAS 3.0 (http://kobas.cbi.pku.edu.cn/kobas3) database. The enriched GO terms and KEGG pathways with corrected *p*-value < 0.05 (FDR) were selected to explore the biological processes. To further clarify the results of the association analysis, significant ROHets were subjected to quantitative trait loci (QTL) enrichment analysis by GALLO package (Genomic Annotation in Livestock for Positional Candidate LOci, https://github.com/pablobio/GALLO) in R software with default setting.

## Results

### Runs of Heterozygosity Detection and Statistics

**FIGURE 1:**
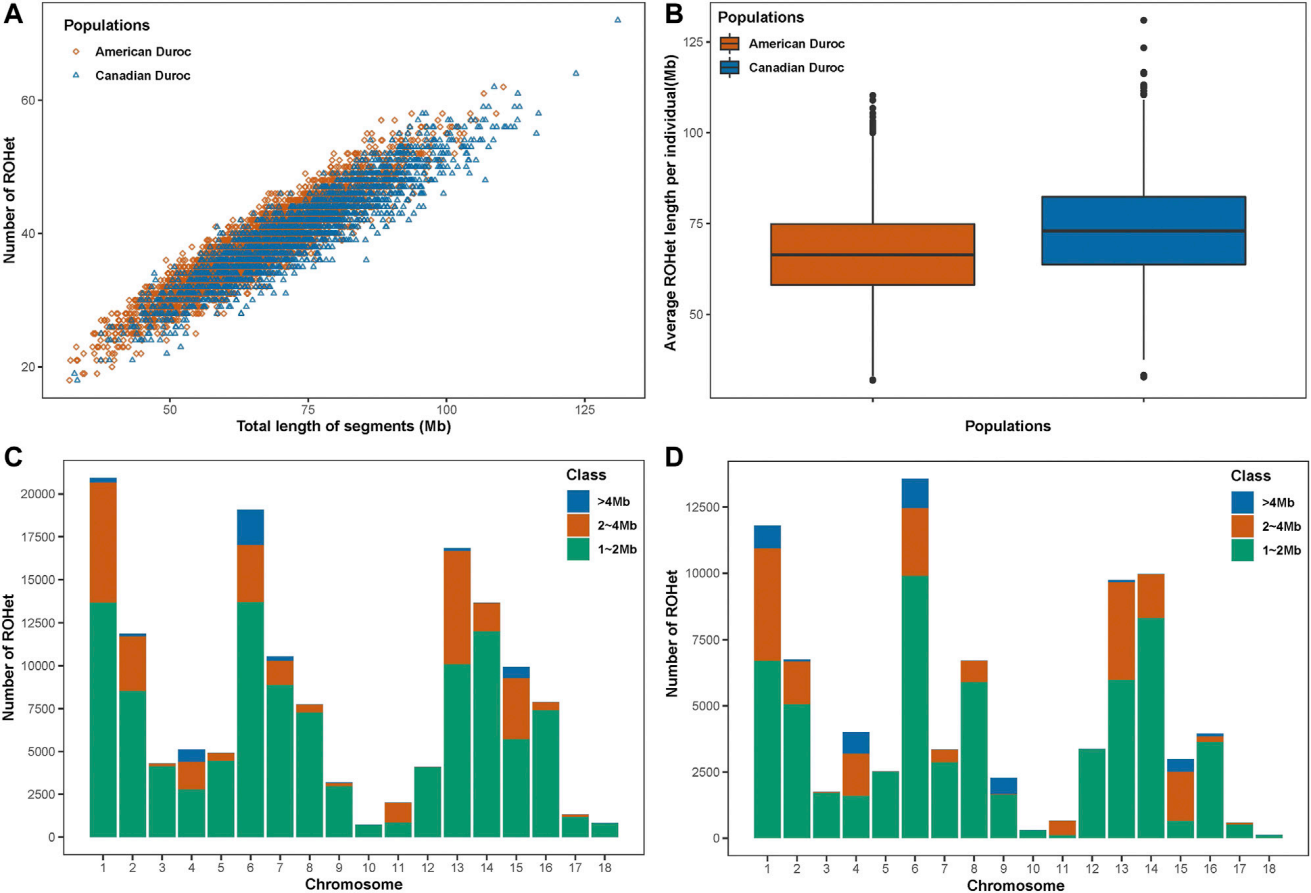
ROHet statistics in two Duroc populations. **(A)** Number and total length of ROHet per individual for two Duroc populations. **(B)** Average ROHet length per individual for two Duroc populations. **(C)** Number of total ROHet and number of ROHet classes per chromosome for the S21 population. **(D)** Number of total ROHet and number of ROHet classes per chromosome for the S22 population.

A total of 145,010 and 84,396 ROHets were detected in the S21 and S22 populations, with an average of 38.46 and 40.26 ROHets for each S21 and S22 pig, respectively (Figure 1A). The mean length of ROHet was 66.53 ± 12.20 Mb (including 22.52 ± 8.56 SNPs) and 73.32 ± 13.77 Mb (including 23.01 ± 8.77 SNPs) in the S21 and S22 populations, respectively (Figure 1B). We found that the $f_{ROHet}$ of S21 (0.027) was significantly (*p* < 0.001) lower than that of S22 (0.030) pigs. As expected, the numbers of short fragments of ROHet (1–2 Mb) accounted for the largest fraction of total ROHet segments in S21 (75.48%) and S22 (72.25%) pigs. The number of ROHet fragments decreased with the increasing of ROHet length (Table 1; Figure 1C and Figure 1D). We further observed that the number (*r* = 0.848, *p* = 8.776 × 10^–6^ in S21; and *r* = 0.796, *p* = 7.787 × 10^–5^ in S22) and genome coverage (*r* = 0.859, *p* = 4.938 × 10^–6^ in S21; and *r* = 0.845, *p* = 1.021 × 10^–5^ in S22) of ROHet on each autosome had significantly positive correlations with chromosome length in S21 and S22 pigs. Chromosomes (SSC) 1 and 6 contained the largest number of ROHet segments in S21 and S22 populations (Figure 1C and Figure 1D), respectively.

**TABLE 1:** Number, percentage, and average length for ROHet categories.

Categories^a^	S21 population			S22 population		
	Count^b^	Percentage^c^ (%)	Average^d^ (Mb)	Count	Percentage (%)	Average (Mb)
1,2 Mb	109,453	75.480	1.369	60,979	72.25	1.374
2–4 Mb	31,307	21.590	2.573	19,338	22.92	2.63
>4 Mb	4,250	2.930	4.812	4,079	4.833	4.673

a Length category of ROHet.

b The number of ROHet, per length category.

c Proportion of the number of ROHet, per length category to total number of ROHet, in the population.

d The average length of ROHet, for each length category.

### ROHet Islands and Gene Annotation

**FIGURE 2:**
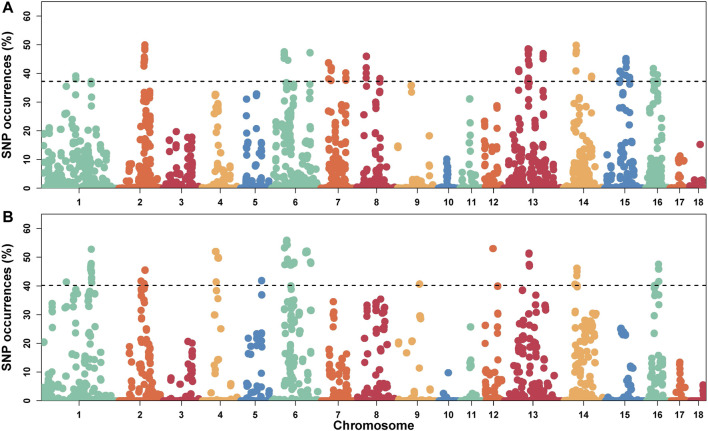
Manhattan plot of the incidence of heterozygotes within ROHets in two Duroc populations. **(A)** The S21 population. **(B)** The S22 population. The dashed lines denote the top 1% threshold.

**FIGURE 3:**
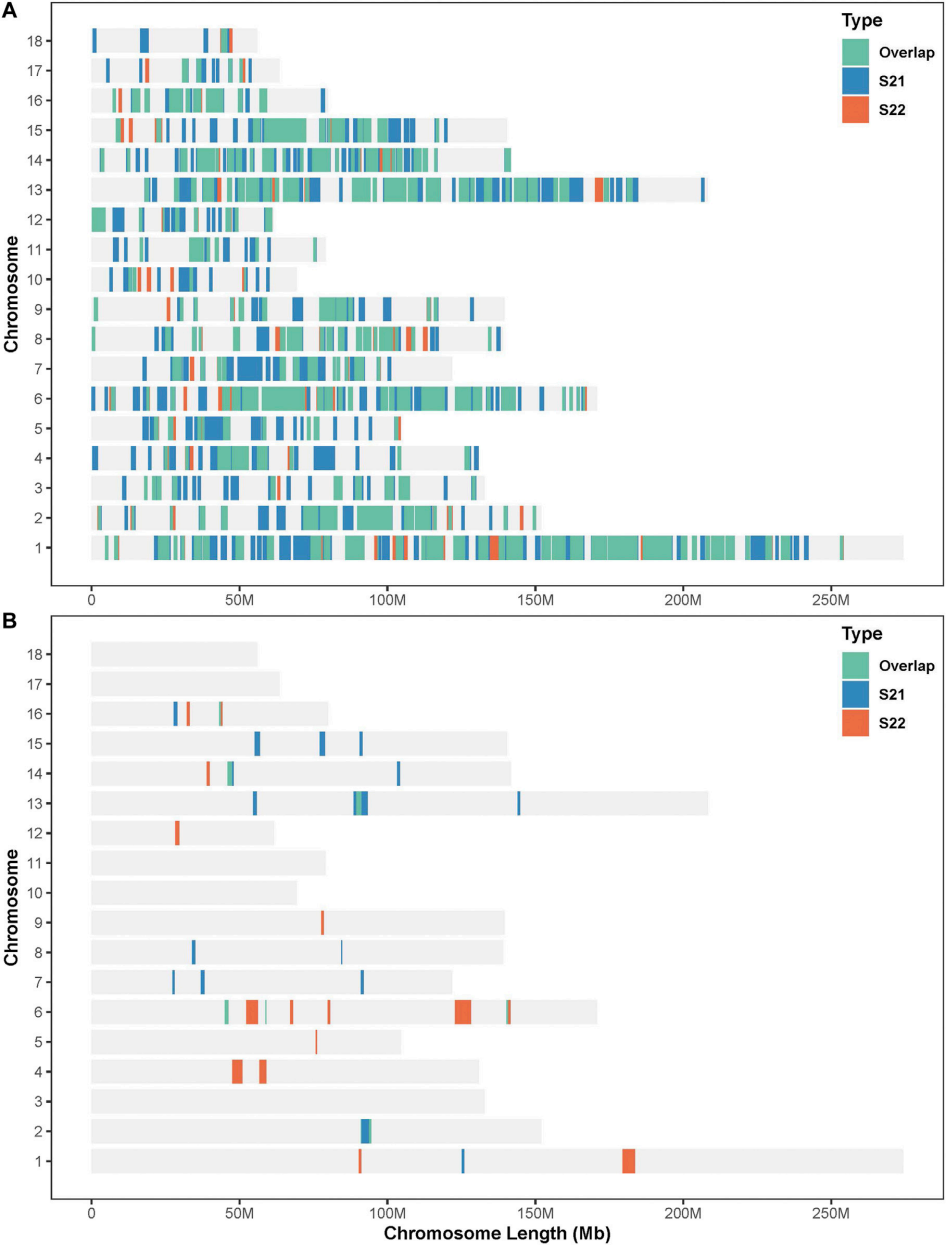
Distribution of the ROHets and ROHet islands in the autosomes of two Duroc populations. **(A)** Distribution of ROHets. **(B)** Distribution of ROHet islands.

In this study, SNPs with top 1% occurrence were identified as ROHet island (Figure2). Figure3A showed the distribution of all ROHets on the genome for two Duroc populations. For S21 pigs, 20 genomic regions located on SSC1, 2, 6, 7, 8, 13, 14, 15, and 16 were detected as ROHet islands (Figure 3B), where a total of 186 genes were annotated. Four GO terms were significantly enriched for these genes, while no KEGG pathway was targeted (Supplementary Table S2). The significant GO terms were involved in *G protein-coupled purinergic nucleotide receptor signaling pathway* (GO: 0035589), *G protein-coupled purinergic nucleotide receptor activity* (GO: 0045028), *odontogenesis of dentin-containing tooth* (GO: 0042475), and *actin cytoskeleton reorganization* (GO: 0031532). For S22 pigs, 23 genomic regions located on SSC1, 2, 4, 5, 6, 9, 12, 13, 14, and 16 were determined to be ROHet islands (Figure 3B), and 317 genes were annotated in these ROHet islands. These genes were significantly enriched in eight GO terms and one KEGG pathway, which were mainly involved in cellular activity and signaling (Supplementary Table S2). Furthermore, the eight overlapping genomic regions of the two Duroc populations were located on SSC2, 6, 13, 14, and 16 with 73 annotated genes. For overlapping ROHet islands, three GO terms and one KEGG pathway were enriched (Supplementary Table S2). These GO terms and KEGG pathways were related to *regulation of transcription, DNA-templated* (GO: 0006355), *negative regulation of canonical Wnt signaling pathway* (GO: 0090090), *JNK cascade* (GO: 0007254), and *Herpes simplex virus 1 infection* (ssc05168).

### Genome-wide ROHet-Traits Association Analysis

**FIGURE 4:**
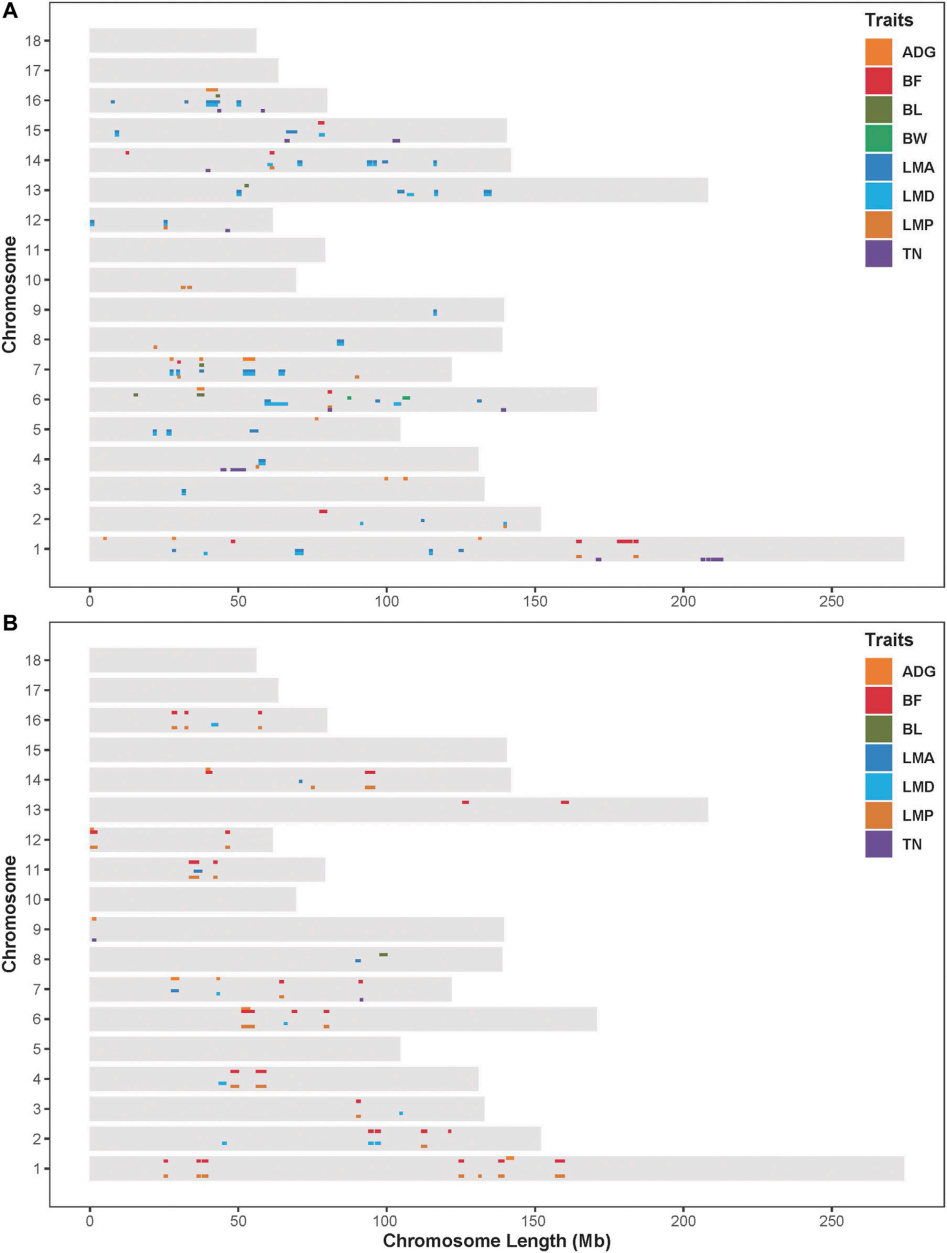
Distribution of significant ROHets in genome-wide ROHet analysis. **(A)** The S21 population. **(B)** The S22 population. ADG, 100 kg average daily gain; BF, 100 kg backfat thickness; BL, body length; BW, birth weight; LMA, loin muscle area; LMD, loin muscle depth; LMP, lean meat percentage; and TN, total teat number.

Results from the association analysis of ROHet in two Duroc populations were reported in Figure 4. For the S21 population, 12, 10, 6, 2, 41, 33, 12, and 14 genome-wide significant ROHets were detected for ADG, BF, BL, BW, LMA, LMD, LMP, and TN traits, respectively (Figure 4A, Supplementary Figure S1). In addition, we identified 35 pleiotropic ROHets (Supplementary Table S3), five of which were significantly associated with three traits, including 80.20–81.57 Mb on SSC6 for BF, LMP, and TN; 26.87–28.12 Mb on SSC7 for ADG, LMA, and LMD; 51.57–55.62 Mb on SSC7 for ADG, LMP, and LMD; 24.88–26.11 Mb on SSC12 for LMA, LMD, and LMP; and 39.21–43.5 Mb on SCC16 for ADG, LMA, and LMD (Figure 4A). Furthermore, eight ROHets were identified on SSC1, 2, 6, 7, 8, 15, and 16, which were overlapped with ROHet islands completely or partly (Table 2). These regions were significantly associated with ADG, BL, BF, LMA, LMD, and TN traits, and 48 genes were annotated (Supplementary Table S4).

**TABLE 2:** Phenotype-related ROHets overlapped with ROHet islands.

Populations	Chr^a^	Regions^b^ (Mb)	Traits^c^	Candidate genes^d^
S21	1	125.113–125.896	LMA	—
	2	91.070–92.080	LMD	—
	6	58.817–59.042	LMA, LMD	—
	7	27.333–28.116	ADG, LMA, LMD	—
	7	36.958–38.156	ADG, BL, LMA	*UBR2*
	8	84.380–84.708	LMA, LMD	*GAB1*
	15	77.157–78.908	BF, LMD	*RAPGEF4*
	16	43.210–43.712	BL, LMA, TN	*CWC27*
S22	2	93.818–94.595	BF, LMD	—
	4	47.639–50.258	BF, LMP	—
	4	56.804–56.958	BF, LMP	—
	4	57.084–59.132	BF, LMP	—
	6	52.311–55.454	ADG, BF, LMP	*CPT1C*, *NR1H2*
	6	79.872–80.604	BF, LMP	—
	14	39.029–39.873	ADG, BF	*TEMP116*
	16	32.246–33.140	BF, LMP	*ITGA2*

a Chr, Chromosome.

b Regions, overlapping regions.

c ADG, 100 kg average daily gain; BF, 100 kg backfat thickness; BL, body length; Candidate genes, gene functions related to the traits under study; LMA, loin muscle area; LMD, loin muscle depth; and LMP, lean meat percentage.

d Gene symbols in italic.

For the S22 population, 8, 34, 1, 4, 8, 27, and 2 genome-wide significant ROHets were detected for ADG, BF, BL, LMA, LMD, LMP, and TN traits, respectively, while no ROHet was significantly associated with BW (Figure 4B, Supplementary Figure S2). Furthermore, 30 pleiotropic ROHets were identified, whereas no ROHet affected three or more traits in the S22 population (Supplementary Table S3). Additionally, we detected eight ROHets located on SSC2, 4, 6, 14, and 16 that were significantly associated with ADG, BF, LMD, and LMP and overlapped with ROHet islands (Table 2). These regions were annotated with 135 genes (Supplementary Table S4).

Pleiotropic ROHets were detected in both Duroc pig populations, possibly due to genetic correlation between traits (Supplementary Table S5). Positive genetic correlation between LMD and LMA in the S21 population (0.989), and negative genetic correlation between BF and LMP in the S22 population (−0.990). Therefore, the significant pleiotropic ROHets identified were mainly associated with LMA and LMD for S21 pigs, and BF and LMP for S22 pigs, respectively. The QTL enrichment analysis found that these significant ROHet regions were mainly enriched in meat and carcass, health, and production traits in two Duroc populations (Supplementary Table S6).

## Discussion

### Distribution of Heterozygous Loci in the Duroc Genome

In this study, we identified ROHet in two Duroc populations and found that the distribution of ROHet island and the $f_{ROHet}$ were different in two Duroc populations. According to our previous study (Zhou et al., 2021), the genetic background of the two Duroc populations are different due to the different intensity and direction of selection, which may lead to diverse heterozygosity-rich regions in the genome. Additionally, Williams et al. (2016) found that heterozygosity-rich regions were not randomly distributed in the Chillingham cattle genome, but rather than clustered in specific chromosomal regions, in analogy to ROH and defined as ROHet. Biscarini et al. (2020) detected three ROHet islands on chromosomes 6, 14, and 21, respectively, in the genome of Maremmana Semi-Feral Cattle. Moreover, a study of 192 Mangalarga Marchador horses (Bizarria Dos Santos et al., 2021) was identified one ROHet island on chromosome 11, and the rest of chromosomes did not form a clear ROHet island. Chillingham cattle, Maremmana Semi-Feral cattle, and Mangalarga Marchador horses were inbred in the previous study, leading to reduced heterozygosity and only a few ROHet islands were formed. In this study, the heterozygous loci were concentrated on several chromosomes in the genome of 5,866 Duroc pigs. ROHet detection results of 5,297 commercial turkeys (*Meleagris gallopavo*) were similar to this study (Marras et al., 2018). A reasonable explanation is that Duroc is also a commercial breed requiring control of the degree of inbreeding, and more heterozygous loci were probably detectable in large populations. On the other hand, the difference in ROHet identification parameters can also have a large impact on the estimation of ROHet (Biscarini et al., 2020). In previous studies in cattle and horses, two or three homozygous genotypes were allowed in a run, whereas in this study only one opposite genotype was allowed. Given the lower inbreeding coefficient of Duroc pigs (Dumitrescu et al., 2005), we set the shortest ROHet length to 1 Mb in order to obtain a longer and more widely distributed ROHet island in the genome. Due to the limitation of chip density, further in-depth study could consider using resequencing data to verify and improve our results.

### ROHet Hotspots Shared by Two Duroc Populations

Individuals with higher heterozygosity potentially have better fitness (Mc Parland et al., 2009). SNP markers covering the genome can provide better insight into the high frequency regions of heterozygosity in the pig genome. The ROHets were shared between Duroc populations of different genetic backgrounds probably related to the survival rate and breed characteristics. In the present study, eight overlapping ROHet islands were identified in two Duroc populations. The *Leukemia Inhibitory Factor* (*LIF*) and *Ewing Sarcoma Breakpoint Region 1* (*EWSR1*) genes were located in the identical region of 46.03–47.41 Mb on SSC14. The *LIF* gene has been reported to be involved in ovulation, early embryonic growth, and implantation in humans (Senturk and Arici, 1998). Tian et al. (2021) demonstrated that *EWSR1* knock out mice caused meiotic arrest. In addition, the *negative regulation of canonical Wnt signaling pathway* term (GO: 0090090) and the Herpes simplex virus 1 *infection pathway* (ssc05168) were significantly enriched (correct *p* < 0.05) in the functional enrichment analysis of the overlapped regions. The *Wnt* signaling is known to be an influential pathway affecting cell proliferation and embryonic development (Muccioli et al., 2021). In addition, it has been suggested that the *negative regulation of canonical Wnt signaling pathway* term may be associated with feed efficiency traits in Nellore cattle (Brunes et al., 2021). The other noteworthy pathway, Herpes simplex virus 1 *infection,* blocks specific immune response through a variety of mechanisms (Eisemann et al., 2007). Hence, high-frequency heterozygous regions in the Duroc genome may enhance fitness and reproductive traits. Duroc pigs are a widely used commercial breed worldwide. The important economic traits of Duroc pigs are affected by strong artificial selection, which can lead to reduced polymorphism in regions near the selected areas due to linkage disequilibrium (LD). Previous literature (Makanjuola et al., 2021; Zhao et al., 2021) reported that some homozygous regions had adverse effects on production and fertility traits in cattle, which may be caused by the increase of unfavorable alleles in LD with benefit alleles during intense selection. Therefore, maintaining genomic heterozygosity within commercial breeds is critical to the sustainability of livestock farming (Williams et al., 2016), and ROHet may be a measure for investigating and managing the genetic diversity of Duroc populations in this study.

### ROHets Significantly Associated With Three Traits

In previous studies, it is possible that there is co-selection between pig traits. Kim et al. (2000) found that backfat thickness and daily weight gain phenotypes in pigs were influenced by different genotypes of the *MC4R* gene. Thus, heterozygous regions within the genome may be able to achieve optimal production performance in Duroc pigs. In the present study, we identified five ROHets that were significantly associated with three traits for the S21 population. In the ROHet of 26.87–28.12 Mb on SSC7 for ADG, LMA, and LMD traits, the *Kelch Like Family Member 31* (*KLHL31*) gene is important in maintaining skeletal muscle architecture, and knock out mice had caused delayed skeletal muscle development (Papizan et al., 2017). In the ROHet of 51.57–55.62 Mb on SSC7 for ADG, LMA, and LMD traits, the *Homer Scaffold Protein 2* (*HOMER2*) gene is involved in osteoclast formation and bone metabolism and its deletion significantly reduces tibial bone density (Son et al., 2019).

In the ROHet of 39.2–43.05 Mb on SSC16, the *Aggrecan* (*ACAN*) gene is known to affect cartilage development, and homozygous and compound heterozygous of the four *ACAN* alleles cause chondrodysplastic dwarfism in Miniature horses (Eberth et al., 2018). In this study, we found that heterozygosity for this genomic region actually led to a reduced phenotype for ADG trait (614.50 g/day for heterozygosity vs. 619.86 g/day for non-heterozygosity, *p* < 0.05). The result revealed that heterozygosity in this segment reduced the growth rate of Duroc pigs. In contrast, heterozygosity for this genomic region caused a significant increase in LMA trait compared to non-heterozygous (39.26 cm^2^ vs. 38.72 cm^2^, *p* < 0.05), while LMD trait were not significantly different (*p* = 0.153). Hedrick (2012) reported that many genes are undergoing heterozygote advantage selection in the genome. However, there was a negative correlation between ADG and LMA in this study (−0.233), showing that ROHet had a positive effect on LMA and an opposite effect on ADG. Therefore, the results confirmed that the genome-wide ROHet analysis could be used to detect relationships between heterozygous regions and phenotypes.

In the ROHet of 39.21–43.05 Mb on SSC16 for ADG, LMA, and LMD traits, *Importin 11* (*IPO11*) gene is involved in and regulates lipid metabolism (Rotroff et al., 2018). In the ROHet of 80.20–81.57 Mb on SSC6, no functional gene was found to be associated with BF, LMP, and TN traits. For the S22 population, it is possible that the number of effective ROHets was too low to detect ROHets that significantly affected the three traits.

### Phenotype-Related ROHets Between Two Populations

In the association analysis, three completely identical ROHet regions were detected in two Duroc populations, including 130.83–131.94 Mb on SSC1, 55.91–56.96 Mb on SSC4, and 31.86–33.14 Mb on SSC16. Notably, these ROHets were significantly associated with different traits in both Duroc populations due to selection disparity in these populations, including carcass, growth, and meat quality traits. Previous studies on commercial pigs using different methods have identified candidate genes for important traits within these ROHet regions. The *Phospholipase C Beta 2* (*PLCB2*) gene was identified by single-step genome-wide association study (ssGWAS) as a candidate gene for the average daily gain trait in Landrace pigs (Hong et al., 2020). Expression of the *Zinc Finger Protein 704* (*ZNF704*) gene played an important role in pork quality traits (Ponsuksili et al., 2014). The *Molybdenum Cofactor Synthesis 2* (*MOCS2*) gene was located in a prominent selective sweep region of the Yorkshire pig genome, enriched in metabolic pathways (Wang et al., 2018).

### Phenotype-Related ROHet Overlapped With ROHet Island

Recent studies have shown that heterozygosity in the genomes of pigs, cattle, and sheep has a positive effect on growth, reproduction, and fitness (Iversen et al., 2019; González-Murray et al., 2020; Cloete et al., 2021). Most of the economic traits in pigs are quantitative traits, controlled by multiple genes. Over 33,000 QTLs have been successfully identified in the pig genome (https://www.animalgenome.org/cgi-bin/QTLdb/index, release 44). If these traits are positively selected (polygenic selection), most favorable alleles in the population may become slightly more frequent. Therefore, we performed association analysis to identify regions of heterozygosity that significantly influence economic traits.

For the S21 population, we detected eight overlapping genome regions between ROHets that were significant in the association analysis and ROHet islands. The genome regions, located in 36.96–38.16 Mb on SSC 7, were associated with BL, LMA, and LMD traits, and *Ubiquitin Protein ligase E3 Component N-Recognin 2* (*UBR2*) gene was harbored. Yang et al. (2014) found that the *UBR2* is in a region of significant differentiation between Chinese indigenous and Western commercial pig breeds. It is possible that Western pigs have a high meat yield and lean meat percentage. The *UBR2* gene was also significantly associated with post-weaning weight gain in sheep (Zhang et al., 2013). Another genome region was associated with LMA and LMD traits, located in 84.40–84.71 Mb on SSC8, including the *GRB2 Associated Binding Protein 1* (*GAB1*) gene. The *GAB1* gene is involved in muscle progenitor cell migration and muscle differentiation pathways (Vasyutina et al., 2005; Koyama et al., 2008), which suggests that *GAB1* may play a role in influencing muscle composition in Duroc pigs. The *Rap Guanine Nucleotide Exchange Factor 4* (*RAPGEF4*) gene located in the region of 77.18–78.91 Mb on SSC15 was associated with BF and LMD traits and was reported to be involved in energy metabolism and differentially expressed in the pectoral muscle of chickens (Lake et al., 2019). The *CWC27 Spliceosome Associated Cyclophilin* (*CWC27*) gene was annotated within the region of 43.21–43.71 Mb on SSC 16 that was associated with BL, LMA, and TN traits. Xu et al. (2017) found that developmental delays in mice with knockout *CWC27* gene.

For the S22 population, eight overlapping regions were detected between ROHet islands and phenotype-related ROHets. *Carnitine palmitoyltransferase 1C* (*CPT1C*) and *Nuclear Receptor Subfamily one Group H Member 2* (*NR1H2*) genes were targeted in the region of 52.31–55.45 Mb on SSC6 which was identified to be associated with BF and LMP traits. The *CPT1C* gene, which encodes the CTP1 enzyme, has differential activity associated with tissue-specific requirements in fatty acid metabolism and energy expenditure, and regulates feed intake leading to obesity (Ramírez et al., 2013; Cirillo et al., 2014). *NR1H2* encodes the liver X receptor, which is an endogenous cholesterol sensor, plays an important role in lipid metabolism, activates the transcription of many genes that are fundamentally involved in lipid metabolism, particularly cholesterol metabolism, and acts as a hypolipidemic agent (Tontonoz and Mangelsdorf, 2003; Mouzat et al., 2011). The *Transmembrane Protein 116* (*TEMP116*) gene was located in 39.03–39.87 Mb on SSC14 and selected as a candidate gene for ADG trait. The *TEMP116* gene was associated with diabetes and its variants could lead to metabolic syndrome (Park et al., 2018). The *Integrin Subunit Alpha 2* (*ITGA2*) gene was located in the region of 32.25–33.14 Mb on SSC16 which was associated with BF and LMP traits. Mutations in the *ITGA2* gene significantly affect total cholesterol, triglycerides, and low-density lipoprotein in humans (Lu et al., 2014).

## Conclusion

This study detected ROHet in the genomes of two Duroc pig populations based on 50 K SNP array and identified the differences in the heterozygosity-rich regions between these two Duroc populations. In addition, genome-wide ROHet analysis revealed that some ROHets were significantly associated with economic traits in pigs. Several overlapping ROHet regions played opposite roles in different traits, revealing the complex genetic basis of heterozygosity. Sixteen promising genes were identified within significant ROHets that regulate growth (*CWC27*, *UBR2*, *ACAN*, *KLHL31,* and *PLCB2*), metabolism (*CPT1C*, *TMEM116*, *NR1H2*, *ITGA2*, *HOMER2*, *IPO11,* and *MOCS2*), and muscle and meat quality (*GAB1*, *RAPGEF4*, *SPOP,* and *ZNF704*) for productive performance. These results will provide new insights and references for pig genetic improvement.

## Data Availability

The datasets presented in this study can be found in online repositories. The names of the repository/repositories and accession number(s) can be found below: https://figshare.com/, https://doi.org/10.6084/m9.figshare.8019551.v1.
