# Supplementary material for: Assessment of Heterozygosity and Genome-Wide Analysis of Heterozygosity Regions in Two Duroc Pig Populations
Source: Front Genet. 2022 Jan 27;12:812456. doi: 10.3389/fgene.2021.812456 (PMC8830653; doi:10.3389/fgene.2021.812456)
Supplement: Supplementary file 1 [file DataSheet1.docx]

**Supplementary files**


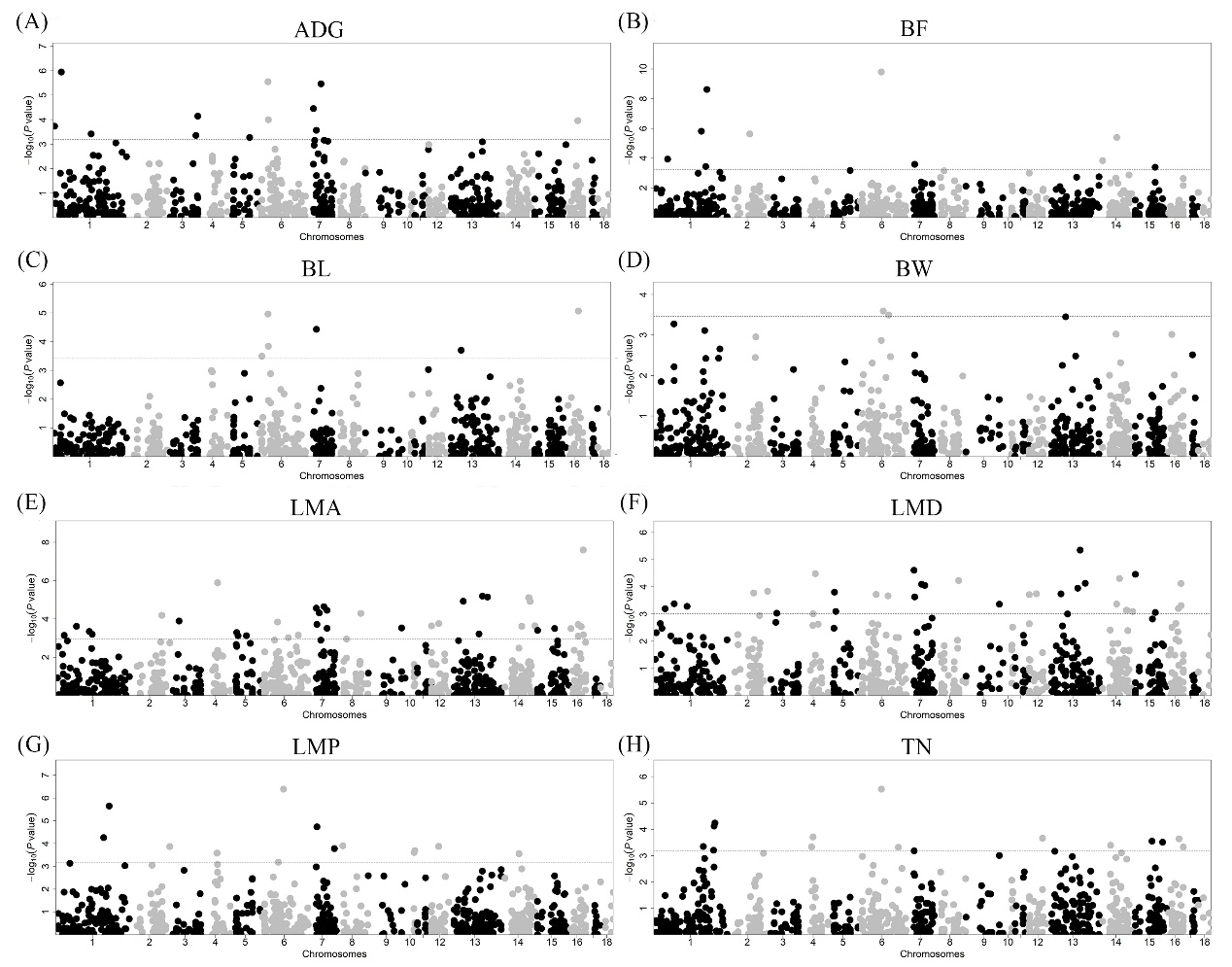


**Supplementary Figure S1** Manhattan plot for genome-wide ROHet analysis in the S21 population. (A) ADG, 100kg average daily gain. (B) BF, 100kg backfat thickness. (C) BL, body length. (D) BW, birth weight. (E) LMA, loin muscle area. (F) LMD, loin muscle depth. (G) LMP, lean meat percentage. (H) TN, total teat number.


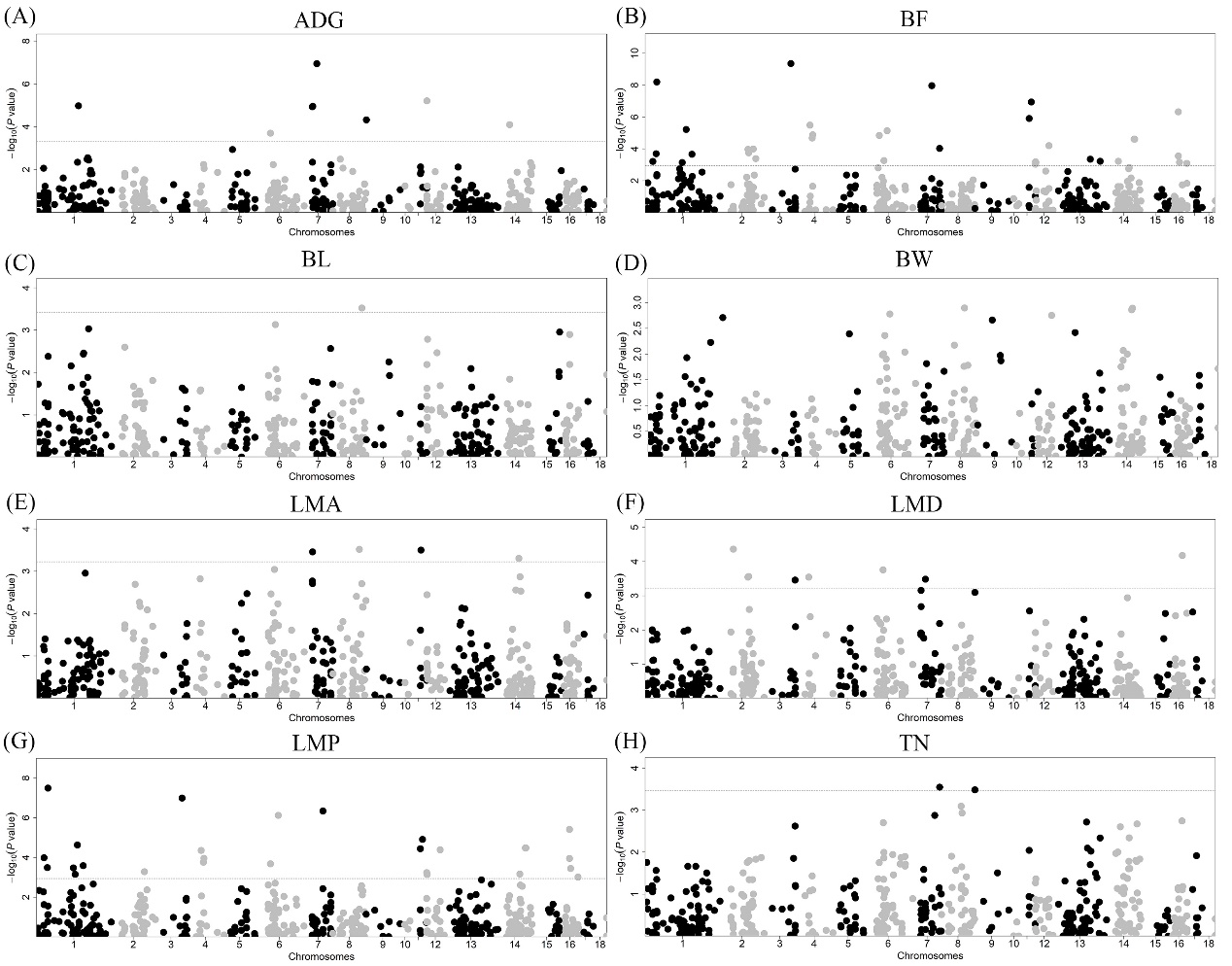


**Supplementary Figure S2** Manhattan plot for genome-wide ROHet analysis in the S22 population. (A) ADG, 100kg average daily gain. (B) BF, 100kg backfat thickness. (C) BL, body length. (D) BW, birth weight. (E) LMA, loin muscle area. (F) LMD, loin muscle depth. (G) LMP, lean meat percentage. (H) TN, total teat number.

**Supplementary Table S1** Descriptive statistics of eight economic traits for two Duroc populations.

| Populations | Traits ^a^ | Count | Mean | SD ^b^ | Max | Min | CV (%) ^c^ |
| --- | --- | --- | --- | --- | --- | --- | --- |
| S21 | ADG | 3770 | 619.43 | 32.7 | 758.54 | 509 | 5.28 |
|  | BF | 3769 | 8.97 | 1.11 | 17.91 | 6.09 | 12.33 |
|  | BL | 3770 | 1.73 | 0.29 | 3.2 | 1 | 16.74 |
|  | BW | 3769 | 10.84 | 1.13 | 16 | 6 | 10.4 |
|  | LMA | 3769 | 62.24 | 1 | 65.27 | 54.3 | 1.61 |
|  | LMD | 3769 | 52.4 | 3.72 | 65 | 37.3 | 7.09 |
|  | LMP | 3769 | 38.77 | 3.51 | 51.56 | 23.69 | 9.05 |
|  | TN | 3768 | 121.3 | 3.51 | 135 | 107 | 2.89 |
| S22 | ADG | 2088 | 613.75 | 43.27 | 769.05 | 478.73 | 7.05 |
|  | BF | 2080 | 9.65 | 1.84 | 17.31 | 5.1 | 19.06 |
|  | BL | 2086 | 122.74 | 3.49 | 138 | 112 | 2.84 |
|  | BW | 2094 | 1.79 | 0.3 | 3 | 1.1 | 16.9 |
|  | LMA | 2080 | 35.81 | 3.62 | 48.84 | 25.4 | 10.12 |
|  | LMD | 2088 | 47.86 | 3.75 | 61.2 | 37 | 7.84 |
|  | LMP | 2080 | 61.02 | 1.52 | 65.06 | 54.93 | 2.48 |
|  | TN | 2094 | 10.9 | 1.16 | 16 | 4 | 10.65 |

^a^ ADG, 100kg average daily gain; BF, 100kg backfat thickness; BL, body length; BW, birth weight; LMA, loin muscle area; LMD, loin muscle depth; LMP, lean meat percentage; TN, total teat number; ^b^ SD, standard deviation; ^c^ CV, coefficient of variation.

**Supplementary Table S2** Gene Ontology (GO) terms and Kyoto Encyclopedia of Genes and Genomes (KEGG) pathways for significant ROHets and overlapping ROHet islands in two Duroc populations (corrected-*P* < 0.05).

| Population | ID | Term | ID | Input number | Background number | P-Value | Corrected P-Value | Input |
| --- | --- | --- | --- | --- | --- | --- | --- | --- |
| S21 | GO:0035589 | G protein-coupled purinergic nucleotide receptor signaling pathway | GO:0035589 | 6 | 14 | 2.24E-09 | 1.95E-06 | *P2RY14, P2RY12, P2RY13, GPR171, P2RY1, GPR87* |
|  | GO:0045028 | G protein-coupled purinergic nucleotide receptor activity | GO:0045028 | 5 | 12 | 5.83E-08 | 2.54E-05 | *GPR171, P2RY14, P2RY13, P2RY12, GPR87* |
|  | GO:0042475 | odontogenesis of dentin-containing tooth | GO:0042475 | 4 | 35 | 0.000110938 | 0.029515843 | *FGF10, NF2, ITGA6, DLX2* |
|  | GO:0031532 | actin cytoskeleton reorganization | GO:0031532 | 4 | 37 | 0.000135239 | 0.029515843 | *FGF10, PLEK2, PTK7, GAB1* |
| S22 | GO:0030141 | secretory granule | GO:0030141 | 9 | 51 | 2.14E-08 | 3.01E-05 | *KLK12, KLK13, KLK10, KLK11, KLK14, KLK4, KLK5, KLK6, KLK8* |
|  | GO:0004252 | serine-type endopeptidase activity | GO:0004252 | 12 | 142 | 1.75E-07 | 0.00012326 | *KLK12, KLK13, KLK10, KLK11, KLK14, KLK15, KLK4, KLK5, KLK6, KLK7, KLK8, RHBDD3* |
|  | GO:0008219 | cell death | GO:0008219 | 4 | 16 | 6.40E-05 | 0.020567708 | *KLK8, NUP62, EMP3, PRKD2* |
|  | GO:0090090 | negative regulation of canonical Wnt signaling pathway | GO:0090090 | 7 | 84 | 7.30E-05 | 0.020567708 | *ZNRF3, SIAH2, RUVBL2, WWTR1, DKKL1, KREMEN1, DACT3* |
|  | GO:0007613 | memory | GO:0007613 | 5 | 41 | 0.000157729 | 0.029391732 | *NTF4, KMT2B, SLC8A2, SYT4, KLK8* |
|  | GO:0005829 | cytosol | GO:0005829 | 42 | 2056 | 0.000164402 | 0.029391732 | *HIF3A, SPHK2, LIF, EHD2, RNF13, ANO6, SAV1, CASTOR1, MOCS2, TEAD2, TXNL4A, DNAAF2, KLHDC1, NAA25, RUVBL2, SELENOW, ARF6, NOP53, POLD1, GRWD1, ZNF260, NF2, CAMTA1, WDR62, AP1B1, FKRP, NLN, VCPKMT, SIAH2, SGTB, PTGIR, ZBTB48, ACOT7, CCDC8, PIK3C3, WWTR1, CABP5, ZNF146, PRKD2, ATF5, SULT2A1, TBC1D10A* |
|  | GO:0007229 | integrin-mediated signaling pathway | GO:0007229 | 6 | 67 | 0.000166998 | 0.029391732 | *ADAMTS6, TYROBP, ITGA1, ITGA2, FERMT2, PTPN11* |
|  | GO:0006508 | proteolysis | GO:0006508 | 13 | 347 | 0.00022554 | 0.035284465 | *ADAMTS6, KLK12, KLK10, KLK11, RHBDD3, KLK14, KLK15, KLK4, KLK5, KLK6, KLK7, KLK8, NLN* |
|  | ssc04392 | Hippo signaling pathway - multiple species | ssc04392 | 5 | 28 | 3.07E-05 | 0.014429418 | *WWTR1, TEAD2, FRMD6, NF2, SAV1* |
| Overlap ROHet islands | GO:0006355 | regulation of transcription, DNA-templated | GO:0006355 | 9 | 458 | 2.09E-06 | 0.000718414 | *KMT2B, ZNF146, ZFP14, TSC22D2, ASCC2, ZNF567, ZNF566, ZNF565, EWSR1* |
|  | GO:0090090 | negative regulation of canonical Wnt signaling pathway | GO:0090090 | 4 | 84 | 6.67E-05 | 0.011470232 | *WWTR1, KREMEN1, ZNRF3, SIAH2* |
|  | GO:0007254 | JNK cascade | GO:0007254 | 2 | 12 | 0.00053217 | 0.04576664 | *CDC42EP5, NPHS1* |
|  | ssc05168 | Herpes simplex virus 1 infection | ssc05168 | 6 | 338 | 0.000202358 | 0.023203754 | *ZNF383, ZNF382, ZFP14, ZNF567, ZNF566, ZNF565* |

**Supplementary Table S3** Pleiotropic ROHets detected by genome-wide ROHets-traits analysis for two Duroc populations.

| Populations | Chr | Strat position (bp) | End position (bp) | Traits |
| --- | --- | --- | --- | --- |
| S21 | 1 | 27750374 | 28912681 | LMA, ADG |
| S21 | 1 | 69057749 | 71984666 | LMA, LMD |
| S21 | 1 | 114290241 | 115553198 | LMA, LMD |
| S21 | 1 | 163890592 | 165732651 | BF, LMP |
| S21 | 1 | 183177984 | 184762708 | BF, LMP |
| S21 | 2 | 139310250 | 140390429 | LMD, LMP |
| S21 | 3 | 30961326 | 32378049 | LMA, LMD |
| S21 | 4 | 56803641 | 59115770 | LMA, LMD |
| S21 | 5 | 21293188 | 22520973 | LMA, LMD |
| S21 | 5 | 25857528 | 27403215 | LMA, LMD |
| S21 | 6 | 36116990 | 37290925 | ADG, BL |
| S21 | 6 | 37350876 | 38471591 | ADG, BL |
| S21 | 6 | 80201311 | 81571496 | BF, LMP, TN |
| S21 | 7 | 26867773 | 28115845 | ADG, LMA, LMD |
| S21 | 7 | 29122742 | 30265671 | LMA, LMD |
| S21 | 7 | 29422512 | 30613007 | BF, LMP |
| S21 | 7 | 36957611 | 38439290 | BL, LMA |
| S21 | 7 | 51570546 | 55617577 | ADG, LMA, LMD |
| S21 | 7 | 63587355 | 65625414 | LMA, LMD |
| S21 | 8 | 83301250 | 85561491 | LMA, LMD |
| S21 | 9 | 115722487 | 116916382 | LMA, LMD |
| S21 | 12 | 24880435 | 26114959 | LMA, LMD, LMP |
| S21 | 13 | 49475808 | 51065807 | LMA, LMD |
| S21 | 13 | 116007043 | 117309989 | LMA, LMD |
| S21 | 13 | 132734637 | 135287248 | LMA, LMD |
| S21 | 14 | 60697353 | 62071531 | BF, LMP |
| S21 | 14 | 69865089 | 71530810 | LMA, LMD |
| S21 | 14 | 93359359 | 95209867 | LMA, LMD |
| S21 | 14 | 95407302 | 96602372 | LMA, LMD |
| S21 | 14 | 115794162 | 116838696 | LMA, LMD |
| S21 | 15 | 8467523 | 9791368 | LMA, LMD |
| S21 | 16 | 39210502 | 43045221 | ADG, LMA, LMD |
| S21 | 16 | 42442998 | 43712400 | BL, LMA |
| S21 | 16 | 49441104 | 50597105 | LMA, LMD |
| S21 | 16 | 49944430 | 50948250 | LMA, LMD |
| S22 | 1 | 24862984 | 26284688 | BF, LMP |
| S22 | 1 | 35899320 | 37261336 | BF, LMP |
| S22 | 1 | 37746276 | 39848331 | BF, LMP |
| S22 | 1 | 124333001 | 125972551 | BF, LMP |
| S22 | 1 | 137678680 | 139712773 | BF, LMP |
| S22 | 1 | 156745942 | 160028398 | BF, LMP |
| S22 | 2 | 93817552 | 95595645 | BF, LMD |
| S22 | 2 | 96104513 | 98041221 | BF, LMD |
| S22 | 2 | 111599367 | 113684705 | BF, LMP |
| S22 | 3 | 89799364 | 91240698 | BF, LMP |
| S22 | 4 | 47410200 | 50257855 | BF, LMP |
| S22 | 4 | 55910511 | 56957663 | BF, LMP |
| S22 | 4 | 57083598 | 59418310 | BF, LMP |
| S22 | 6 | 51038832 | 55453781 | BF, LMP |
| S22 | 6 | 78887635 | 80603591 | BF, LMP |
| S22 | 7 | 42731709 | 43839663 | ADG, LMD |
| S22 | 7 | 63885883 | 65356760 | BF, LMP |
| S22 | 9 | 813959 | 2069497 | ADG, TN |
| S22 | 11 | 33429680 | 36712215 | BF, LMP |
| S22 | 11 | 41678741 | 42958919 | BF, LMP |
| S22 | 12 | 94345 | 1280066 | ADG, BF |
| S22 | 12 | 94345 | 1374330 | BF, LMP |
| S22 | 12 | 1490573 | 2531698 | BF, LMP |
| S22 | 12 | 45675657 | 47184260 | BF, LMP |
| S22 | 14 | 92785105 | 94755484 | BF, LMP |
| S22 | 14 | 94798669 | 96110724 | BF, LMP |
| S22 | 16 | 27608306 | 29352738 | BF, LMP |
| S22 | 16 | 27743279 | 29252377 | BF, LMP |
| S22 | 16 | 31864109 | 33139580 | BF, LMP |
| S22 | 16 | 56756857 | 57946318 | BF, LMP |

**Supplementary Table S4** Genome-wide significant ROHets overlapped with ROHet islands.

| Populations | Chr | Position (bp) | Trait | Genes |
| --- | --- | --- | --- | --- |
| S21 | 1 | 125112608-125895939 | LMA | *-* |
| S21 | 2 | 91070076-92080173 | LMD | *VCAN, HAPLN1* |
| S21 | 6 | 58817365-59042429 | LMA, LMD | *CDC42EP5, LENG9, LENG8, TTYH1* |
| S21 | 7 | 27333265-28115845 | ADG, LMA, LMD | *KHDRBS2* |
| S21 | 7 | 36957611-38156399 | ADG, BL, LMA | *FRS3, TOMM6, USP49, CCND3, TAF8, C6orf132, GUCA1A, GUCA1B, MRPS10, TRERF1,* ***UBR2****, PRPH2, BICRAL, CNPY3, PEX6, PPP2R5D MEA1, KLHDC3, CUL7, KLC4, PTK7* |
| S21 | 8 | 84379833-84708131 | LMA, LMD | ***GAB1****, USP38* |
| S21 | 15 | 77157077-78908165 | BF, LMD | *TLK1, METTL8, DCAF17, CYBRD1, SLC25A12, HAT1, METAP1D, DLX1, DLX2, ITGA6, PDK1,* ***RAPGEF4*** |
| S21 | 16 | 43210166-43712400 | BL, LMA, TN | *SHISAL2B, SREK1IP1,* ***CWC27****, ADAMTS6* |
| S22 | 2 | 93817552-94595399 | BF, LMD | *COX7C* |
| S22 | 4 | 47639103-50257855 | BF, LMP | *MMP16, CNBD1, CNGB3* |
| S22 | 4 | 56803641-56957663 | BF, LMP | *HEY1, STMN2* |
| S22 | 4 | 57083598-59132370 | BF, LMP | *IL7, ZC2HC1A* |
| S22 | 6 | 52311424-55453781 | ADG, BF, LMP | *CCDC61, HIF3A, PPP5C, CCDC8, PTGIR, GNG8, DACT3, PRKD2, STRN4, FKRP, SLC1A5, AP2S1, ARHGAP35, NPAS1, TMEM160, ZC3H4, SAE1, CCDC9, INAFM1, C5AR2, DHX34, MEIS3, SLC8A2, KPTN, ZNF541, BICRA, EHD2, NOP53, SELENOW, CRX, SULT2A1, BSPH1, ELSPBP1, CABP5, LIG1, ZSWIM9, EMP3, TMEM143, SYNGR4, GRIN2D, GRWD1, KCNJ14, CYTH2, LMTK3, FAM83E, RPL18, SPHK2, DBP, CA11, NTN5, MAMSTR, IZUMO1, RASIP1, BCAT2, PLEKHA4, PPP1R15A, TULP2, NUCB1, RUVBL2, NTF4, KCNA7, SNRNP70, PPFIA3, HRC, TRPM4, CD37, TEAD2, DKKL1, KASH5, PTH2, GFY, SLC17A7, PIH1D1, ALDH16A1, FCGRT, RCN3, PRRG2, NOSIP, PRR12, RRAS, SCAF1, BCL2L12, PRMT1,* ***CPT1C****, TSKS, AP2A1, MED25, FUZ, PTOV1, PNKP, AKT1S1, TBC1D17, IL4I1, NUP62, ATF5, VRK3, ZNF473, IZUMO2, MYH14, KCNC3, NAPSA,* ***NR1H2****, POLD1, MYBPC2, FAM71E1, EMC10, LRRC4B, SYT3* |
| S22 | 6 | 79872328-80603591 | BF, LMP | *HSPG2, CDC42, ZBTB40, EPHA8, C1QA, C1QC, C1QB* |
| S22 | 14 | 39029446-39873272 | ADG, BF | *RPH3A, PTPN11, HECTD4, TRAFD1, NAA25, ERP29,* ***TMEM116*** |
| S22 | 16 | 32246012-33139580 | BF, LMP | *ITGA1,* ***ITGA2****, MOCS2, NDUFS4, ARL15* |
| Position, overlapping position; Genes, genes were annotation within overlapping regions; Genes in bold are candidate for growth traits in present study. | | | | |

**Supplementary Table S5** Genetic correlations between eight economic traits in two Durocs.

| Traits | ADG | BF | BL | BW | LMA | LMP | LMD | TN |
| --- | --- | --- | --- | --- | --- | --- | --- | --- |
| ADG |  | -0.17159 | 0.8445 | 0.36587 | -0.23283 | 0.33831 | 0.33467 | -0.11744 |
| BF | 0.07394 |  | -0.28441 | -0.13891 | 0.17467 | -0.99319 | 0.12794 | 0.51648 |
| BL | 0.77223 | 0.20621 |  | 0.56282 | 0.17025 | 0.05886 | 0.57232 | 0.04035 |
| BW | 0.49014 | -0.13523 | 0.35432 |  | -0.05512 | 0.13692 | 0.03845 | 0.42885 |
| LMA | -0.08119 | -0.09752 | -0.45698 | 0.13003 |  | 0.62225 | 0.98856 | -0.0725 |
| LMP | 0.20501 | -0.9896 | 0.33618 | 0.1731 | 0.80361 |  | 0.89134 | 0.08663 |
| LMD | 0.68259 | 0.09397 | 0.2129 | 0.14196 | 0.9971 | 0.70777 |  | -0.13535 |
| TN | 0.0367 | -0.1459 | -0.19853 | 0.05176 | -0.1031 | -0.50741 | -0.15614 |  |
| Estimation of genetic correlation for S21 below the diagonal and genetic correlation for S22 above the diagonal. | | | | | | | | |

**Supplementary Table S6** QTL enrichment analysis of significant ROHet in genome-wide ROHet analysis (corrected-P < 0.05).

| Populations | Traits | QTL | N_QTLs | N_QTLs_db | pvalue | adj.pval | QTL_type |
| --- | --- | --- | --- | --- | --- | --- | --- |
| S21 | ADG | Adipocyte diameter | 16 | 66 | 1.22E-08 | 6.52E-07 | Meat and Carcass |
| S21 | ADG | Arachidic acid content | 10 | 39 | 3.90E-06 | 0.000129998 | Meat and Carcass |
| S21 | ADG | Arachidic acid to stearic acid ratio | 14 | 121 | 0.000651394 | 0.009153797 | Meat and Carcass |
| S21 | ADG | Average backfat thickness | 40 | 437 | 5.40E-06 | 0.000133275 | Meat and Carcass |
| S21 | ADG | backfat above muscle dorsi | 7 | 26 | 8.06E-05 | 0.001435409 | Meat and Carcass |
| S21 | ADG | Backfat at last rib | 28 | 268 | 1.26E-05 | 0.000257994 | Meat and Carcass |
| S21 | ADG | backfat at mid-back | 9 | 32 | 5.18E-06 | 0.000133275 | Meat and Carcass |
| S21 | ADG | Backfat weight | 9 | 43 | 6.82E-05 | 0.00130142 | Meat and Carcass |
| S21 | ADG | Base excess | 3 | 7 | 0.002371515 | 0.0275302 | Health |
| S21 | ADG | Belly meat content | 3 | 9 | 0.005338083 | 0.049147178 | Meat and Carcass |
| S21 | ADG | Cis-11-Eicosenoic acid to oleic acid ratio | 7 | 42 | 0.001826064 | 0.022161776 | Meat and Carcass |
| S21 | ADG | Conductivity 24 hours post-mortem | 6 | 36 | 0.003837919 | 0.039412473 | Meat and Carcass |
| S21 | ADG | Creatinine level | 4 | 15 | 0.003066542 | 0.034115276 | Health |
| S21 | ADG | Ear erectness | 8 | 25 | 5.99E-06 | 0.000133275 | Exterior |
| S21 | ADG | Ear weight | 9 | 21 | 8.29E-08 | 3.16E-06 | Exterior |
| S21 | ADG | Eicosenoic acid to eicosanoic acid ratio | 9 | 51 | 0.000271215 | 0.004525897 | Meat and Carcass |
| S21 | ADG | Feet and leg conformation | 2 | 3 | 0.00528432 | 0.049147178 | Exterior |
| S21 | ADG | Feet weight | 3 | 5 | 0.000722664 | 0.009647565 | Meat and Carcass |
| S21 | ADG | Head weight | 201 | 351 | 5.20E-183 | 1.39E-180 | Meat and Carcass |
| S21 | ADG | Leaf fat weight | 6 | 37 | 0.004418476 | 0.043693818 | Meat and Carcass |
| S21 | ADG | Lip thickness | 21 | 21 | 1.40E-29 | 1.87E-27 | Meat and Carcass |
| S21 | ADG | Liver weight | 6 | 29 | 0.001205951 | 0.015332805 | Meat and Carcass |
| S21 | ADG | Loin fat percentage | 4 | 9 | 0.000347481 | 0.005457496 | Meat and Carcass |
| S21 | ADG | Loin muscle area | 57 | 401 | 1.47E-15 | 1.30E-13 | Meat and Carcass |
| S21 | ADG | Mouth width, including lips | 10 | 10 | 1.90E-14 | 1.27E-12 | Meat and Carcass |
| S21 | ADG | NADP-malate dehydrogenase activity | 7 | 10 | 2.68E-08 | 1.19E-06 | Meat and Carcass |
| S21 | ADG | NADPH-generating enzyme activity | 4 | 10 | 0.000559605 | 0.008300807 | Meat and Carcass |
| S21 | ADG | Physis score | 3 | 8 | 0.003674549 | 0.039244183 | Production |
| S21 | ADG | Shoulder subcutaneous fat thickness | 13 | 69 | 5.98E-06 | 0.000133275 | Meat and Carcass |
| S21 | BF | Abdominal fat weight | 9 | 40 | 9.25E-05 | 0.001141023 | Meat and Carcass |
| S21 | BF | Average backfat thickness | 42 | 437 | 1.66E-05 | 0.000285903 | Meat and Carcass |
| S21 | BF | Backfat at first rib | 9 | 54 | 0.000985311 | 0.008506523 | Meat and Carcass |
| S21 | BF | Backfat at last lumbar | 8 | 58 | 0.006211488 | 0.041250651 | Meat and Carcass |
| S21 | BF | Backfat at last rib | 23 | 268 | 0.005254923 | 0.035816449 | Meat and Carcass |
| S21 | BF | backfat at mid-back | 8 | 32 | 0.000102452 | 0.001206134 | Meat and Carcass |
| S21 | BF | Backfat weight | 7 | 43 | 0.004060079 | 0.029210016 | Meat and Carcass |
| S21 | BF | Body height | 12 | 34 | 2.88E-08 | 1.07E-06 | Production |
| S21 | BF | body length | 13 | 79 | 9.20E-05 | 0.001141023 | Production |
| S21 | BF | Cannon bone circumference | 23 | 75 | 4.09E-13 | 2.65E-11 | Exterior |
| S21 | BF | Carcass weight (cold) | 8 | 42 | 0.000751088 | 0.007204881 | Meat and Carcass |
| S21 | BF | Carcass weight (hot) | 13 | 85 | 0.000197401 | 0.002222904 | Meat and Carcass |
| S21 | BF | Conformation score | 5 | 22 | 0.00334087 | 0.02472244 | Exterior |
| S21 | BF | Creatine kinase level | 4 | 13 | 0.002653194 | 0.020342229 | Health |
| S21 | BF | Creatinine level | 4 | 15 | 0.004691152 | 0.032838063 | Health |
| S21 | BF | External fat on ham | 6 | 25 | 0.000967867 | 0.008506523 | Meat and Carcass |
| S21 | BF | Facial morphology | 8 | 13 | 2.84E-08 | 1.07E-06 | Exterior |
| S21 | BF | Fat area percentage in carcass | 12 | 34 | 2.88E-08 | 1.07E-06 | Meat and Carcass |
| S21 | BF | Fat to meat ratio | 5 | 17 | 0.000958448 | 0.008506523 | Meat and Carcass |
| S21 | BF | Femur length | 12 | 45 | 9.26E-07 | 2.40E-05 | Production |
| S21 | BF | Head weight | 201 | 351 | 3.40E-172 | 8.79E-170 | Meat and Carcass |
| S21 | BF | Hemoglobin | 9 | 62 | 0.002670408 | 0.020342229 | Health |
| S21 | BF | Hip bone length | 7 | 11 | 1.60E-07 | 5.17E-06 | Production |
| S21 | BF | Humerus length | 12 | 52 | 4.87E-06 | 0.000105103 | Production |
| S21 | BF | Leaf fat weight | 9 | 37 | 4.79E-05 | 0.000730038 | Meat and Carcass |
| S21 | BF | Linolenic acid content | 6 | 36 | 0.006794729 | 0.043995871 | Meat and Carcass |
| S21 | BF | Lip thickness | 21 | 21 | 1.71E-28 | 2.21E-26 | Meat and Carcass |
| S21 | BF | Liver weight | 7 | 29 | 0.000352672 | 0.003513158 | Meat and Carcass |
| S21 | BF | Loin fat percentage | 3 | 9 | 0.007425877 | 0.046909809 | Meat and Carcass |
| S21 | BF | Loin muscle area | 38 | 401 | 5.55E-05 | 0.000756491 | Meat and Carcass |
| S21 | BF | Loin muscle depth | 17 | 90 | 1.14E-06 | 2.68E-05 | Meat and Carcass |
| S21 | BF | Mean corpuscular hemoglobin concentration | 30 | 312 | 0.000250662 | 0.002695949 | Health |
| S21 | BF | Meat color-a | 6 | 20 | 0.000260227 | 0.002695949 | Meat and Carcass |
| S21 | BF | Mouth width, including lips | 10 | 10 | 6.21E-14 | 5.36E-12 | Meat and Carcass |
| S21 | BF | NADP-malate dehydrogenase activity | 5 | 10 | 5.17E-05 | 0.000744296 | Meat and Carcass |
| S21 | BF | pH for Semimembranosus | 10 | 38 | 8.56E-06 | 0.000158398 | Meat and Carcass |
| S21 | BF | Scapula length | 6 | 29 | 0.002203652 | 0.017835812 | Production |
| S21 | BF | Shoulder subcutaneous fat thickness | 13 | 69 | 2.10E-05 | 0.000340071 | Meat and Carcass |
| S21 | BF | Skin thickness | 4 | 11 | 0.001322554 | 0.011049727 | Meat and Carcass |
| S21 | BF | Tibia length | 12 | 54 | 7.41E-06 | 0.000147555 | Production |
| S21 | BF | Ulna length | 12 | 45 | 9.26E-07 | 2.40E-05 | Production |
| S21 | BL | Adipocyte diameter | 14 | 66 | 1.78E-11 | 1.77E-09 | Meat and Carcass |
| S21 | BL | Alkaline phosphatase activity | 5 | 50 | 0.002403497 | 0.023914794 | Health |
| S21 | BL | Average backfat thickness | 33 | 437 | 1.61E-11 | 1.77E-09 | Meat and Carcass |
| S21 | BL | Average daily gain | 31 | 734 | 2.71E-05 | 0.000771408 | Production |
| S21 | BL | backfat above muscle dorsi | 5 | 26 | 0.000107923 | 0.002386308 | Meat and Carcass |
| S21 | BL | Backfat at last lumbar | 6 | 58 | 0.000748289 | 0.011454579 | Meat and Carcass |
| S21 | BL | Backfat at last rib | 23 | 268 | 1.75E-09 | 1.16E-07 | Meat and Carcass |
| S21 | BL | backfat at mid-back | 4 | 32 | 0.002896402 | 0.027446858 | Meat and Carcass |
| S21 | BL | Backfat weight | 7 | 43 | 1.41E-05 | 0.000473651 | Meat and Carcass |
| S21 | BL | cis-9-Heptadecenoic acid content | 2 | 5 | 0.003378402 | 0.029230523 | Meat and Carcass |
| S21 | BL | Conductivity 24 hours post-mortem | 5 | 36 | 0.000530097 | 0.008790773 | Meat and Carcass |
| S21 | BL | Creatine kinase level | 3 | 13 | 0.001628613 | 0.021497453 | Health |
| S21 | BL | Ear weight | 5 | 21 | 3.61E-05 | 0.000897313 | Exterior |
| S21 | BL | Fat-cuts percentage | 6 | 44 | 0.000162781 | 0.003239337 | Meat and Carcass |
| S21 | BL | Lactate dehydrogenase level | 2 | 4 | 0.002052521 | 0.021497453 | Health |
| S21 | BL | Loin and ham percentage in carcass | 3 | 8 | 0.000341995 | 0.006187001 | Meat and Carcass |
| S21 | BL | Loin fat percentage | 4 | 9 | 1.43E-05 | 0.000473651 | Meat and Carcass |
| S21 | BL | Meat color-a | 3 | 20 | 0.005889389 | 0.048832848 | Meat and Carcass |
| S21 | BL | NADP-malate dehydrogenase activity | 5 | 10 | 5.30E-07 | 2.64E-05 | Meat and Carcass |
| S21 | BL | Shoulder external fat weight | 4 | 28 | 0.001749455 | 0.021497453 | Meat and Carcass |
| S21 | BL | Shoulder subcutaneous fat thickness | 6 | 69 | 0.001864471 | 0.021497453 | Meat and Carcass |
| S21 | BL | Subcutanous fat area | 4 | 28 | 0.001749455 | 0.021497453 | Meat and Carcass |
| S21 | BL | subjective pork flavor in lean | 2 | 4 | 0.002052521 | 0.021497453 | Meat and Carcass |
| S21 | BL | Time spent socializing | 2 | 5 | 0.003378402 | 0.029230523 | Exterior |
| S21 | BW | Backfat at last rib | 18 | 268 | 1.51E-11 | 7.68E-10 | Meat and Carcass |
| S21 | BW | Backfat at tenth rib | 13 | 221 | 5.19E-08 | 1.77E-06 | Meat and Carcass |
| S21 | BW | Empty body lipid content | 3 | 13 | 0.000155654 | 0.00264611 | Meat and Carcass |
| S21 | BW | Fat area percentage in carcass | 12 | 34 | 4.20E-17 | 4.28E-15 | Meat and Carcass |
| S21 | BW | Fat weight (total) | 3 | 15 | 0.000244582 | 0.003563904 | Meat and Carcass |
| S21 | BW | Ham weight | 8 | 129 | 1.37E-05 | 0.000279489 | Meat and Carcass |
| S21 | BW | Leptin level | 2 | 6 | 0.001023431 | 0.011598887 | Health |
| S21 | BW | Loin fat percentage | 2 | 9 | 0.002415814 | 0.020534423 | Meat and Carcass |
| S21 | BW | Loin muscle area | 14 | 401 | 8.26E-06 | 0.000210716 | Meat and Carcass |
| S21 | BW | Meat color a* | 6 | 188 | 0.005171055 | 0.037674831 | Meat and Carcass |
| S21 | BW | muscle protein percentage | 3 | 29 | 0.001801518 | 0.016704981 | Meat and Carcass |
| S21 | BW | pH for Semimembranosus | 3 | 38 | 0.003935367 | 0.030877494 | Meat and Carcass |
| S21 | BW | Stiffness | 2 | 5 | 0.000686077 | 0.00874748 | Meat and Carcass |
| S21 | BW | Thoracic vertebra number | 4 | 60 | 0.001616757 | 0.016490926 | Exterior |
| S21 | LMA | Abdominal fat weight | 15 | 40 | 2.53E-06 | 5.65E-05 | Meat and Carcass |
| S21 | LMA | Actinobacillus pleuropneumoniae susceptibility | 15 | 78 | 0.007873476 | 0.049785452 | Health |
| S21 | LMA | Adipocyte diameter | 30 | 66 | 6.87E-14 | 5.22E-12 | Meat and Carcass |
| S21 | LMA | androstenone, laboratory | 78 | 111 | 4.11E-53 | 5.21E-51 | Meat and Carcass |
| S21 | LMA | Arachidic acid content | 16 | 39 | 2.75E-07 | 6.98E-06 | Meat and Carcass |
| S21 | LMA | Arachidic acid to stearic acid ratio | 28 | 121 | 1.20E-05 | 0.000217217 | Meat and Carcass |
| S21 | LMA | Aspartate aminotransferase activity | 14 | 22 | 1.04E-09 | 3.95E-08 | Health |
| S21 | LMA | Average backfat thickness | 63 | 437 | 0.001122503 | 0.011848641 | Meat and Carcass |
| S21 | LMA | backfat above muscle dorsi | 9 | 26 | 0.000531071 | 0.006726897 | Meat and Carcass |
| S21 | LMA | Backfat at first rib | 12 | 54 | 0.005132058 | 0.039336349 | Meat and Carcass |
| S21 | LMA | Backfat at last rib | 61 | 268 | 2.59E-10 | 1.23E-08 | Meat and Carcass |
| S21 | LMA | backfat at mid-back | 16 | 32 | 8.55E-09 | 2.71E-07 | Meat and Carcass |
| S21 | LMA | backfat at P2 position | 6 | 12 | 0.000473368 | 0.00620275 | Meat and Carcass |
| S21 | LMA | Backfat at tenth rib | 42 | 221 | 2.01E-05 | 0.000335129 | Meat and Carcass |
| S21 | LMA | Backfat weight | 10 | 43 | 0.00733049 | 0.049742613 | Meat and Carcass |
| S21 | LMA | Basophil number | 87 | 102 | 3.03E-72 | 5.75E-70 | Health |
| S21 | LMA | Belly weight | 8 | 30 | 0.006722853 | 0.047081566 | Meat and Carcass |
| S21 | LMA | Blood non-esterified fatty acid level | 9 | 11 | 3.65E-08 | 9.91E-07 | Health |
| S21 | LMA | body weight (17 weeks) | 4 | 8 | 0.004596748 | 0.036390919 | Production |
| S21 | LMA | Body weight (20 weeks) | 3 | 5 | 0.007991875 | 0.049785452 | Production |
| S21 | LMA | Body weight (34 weeks) | 5 | 12 | 0.003890178 | 0.031867338 | Production |
| S21 | LMA | Body weight (end of test) | 6 | 12 | 0.000473368 | 0.00620275 | Production |
| S21 | LMA | C3c concentration | 10 | 33 | 0.000871497 | 0.009740261 | Health |
| S21 | LMA | Carcass length | 31 | 197 | 0.005346317 | 0.039336349 | Meat and Carcass |
| S21 | LMA | Carcass weight (hot) | 23 | 85 | 4.53E-06 | 9.56E-05 | Meat and Carcass |
| S21 | LMA | Conductivity 45 minutes post-mortem | 32 | 201 | 0.003941487 | 0.031867338 | Meat and Carcass |
| S21 | LMA | Creatine kinase level | 6 | 13 | 0.000806536 | 0.00928739 | Health |
| S21 | LMA | Creatinine level | 6 | 15 | 0.00198115 | 0.019303513 | Health |
| S21 | LMA | Diameter of type I muscle fibers | 4 | 9 | 0.00763793 | 0.049785452 | Meat and Carcass |
| S21 | LMA | Ear erectness | 15 | 25 | 8.52E-10 | 3.60E-08 | Exterior |
| S21 | LMA | Ear weight | 9 | 21 | 7.85E-05 | 0.001242492 | Exterior |
| S21 | LMA | Eicosenoic acid to eicosanoic acid ratio | 12 | 51 | 0.003122938 | 0.028255149 | Meat and Carcass |
| S21 | LMA | Enterotoxigenic E. coli susceptibility | 34 | 125 | 2.30E-08 | 6.73E-07 | Health |
| S21 | LMA | Feet weight | 4 | 5 | 0.000418185 | 0.005885561 | Meat and Carcass |
| S21 | LMA | Ham fat thickness | 5 | 12 | 0.003890178 | 0.031867338 | Meat and Carcass |
| S21 | LMA | Ham weight | 22 | 129 | 0.006814437 | 0.047081566 | Meat and Carcass |
| S21 | LMA | Head weight | 205 | 351 | 1.04E-114 | 3.97E-112 | Meat and Carcass |
| S21 | LMA | Hemoglobin | 14 | 62 | 0.002226823 | 0.021154823 | Health |
| S21 | LMA | Interleukin-10 level | 8 | 25 | 0.001930481 | 0.019303513 | Health |
| S21 | LMA | Intermuscular fat content | 13 | 51 | 0.000971425 | 0.010546904 | Meat and Carcass |
| S21 | LMA | Lactate dehydrogenase level | 3 | 4 | 0.003447661 | 0.030467701 | Health |
| S21 | LMA | Leaf fat weight | 12 | 37 | 0.00013203 | 0.002006856 | Meat and Carcass |
| S21 | LMA | Lip thickness | 21 | 21 | 5.67E-22 | 5.38E-20 | Meat and Carcass |
| S21 | LMA | Liver weight | 8 | 29 | 0.005382869 | 0.039336349 | Meat and Carcass |
| S21 | LMA | Loin fat percentage | 5 | 9 | 0.00079527 | 0.00928739 | Meat and Carcass |
| S21 | LMA | Loin muscle area | 81 | 401 | 1.94E-10 | 1.05E-08 | Meat and Carcass |
| S21 | LMA | Mouth width, including lips | 10 | 10 | 7.76E-11 | 4.92E-09 | Meat and Carcass |
| S21 | LMA | Muscle moisture percentage | 20 | 87 | 0.000221586 | 0.003238561 | Meat and Carcass |
| S21 | LMA | muscle protein percentage | 8 | 29 | 0.005382869 | 0.039336349 | Meat and Carcass |
| S21 | LMA | NADP-malate dehydrogenase activity | 7 | 10 | 7.72E-06 | 0.000146729 | Meat and Carcass |
| S21 | LMA | pH 24 hr post-mortem (loin) | 40 | 252 | 0.001474772 | 0.015146307 | Meat and Carcass |
| S21 | LMA | pH for Semimembranosus | 14 | 38 | 6.95E-06 | 0.000138922 | Meat and Carcass |
| S21 | LMA | Salmonella count in liver and spleen | 5 | 12 | 0.003890178 | 0.031867338 | Health |
| S21 | LMA | Shoulder subcutaneous fat thickness | 22 | 69 | 3.41E-07 | 8.09E-06 | Meat and Carcass |
| S21 | LMA | Shoulder weight | 30 | 96 | 4.55E-09 | 1.57E-07 | Meat and Carcass |
| S21 | LMA | Skin percentage | 3 | 5 | 0.007991875 | 0.049785452 | Meat and Carcass |
| S21 | LMA | Skin thickness | 5 | 11 | 0.002466507 | 0.022860312 | Meat and Carcass |
| S21 | LMA | Testicular percentage | 6 | 8 | 2.03E-05 | 0.000335129 | Reproduction |
| S21 | LMA | Testicular weight | 7 | 17 | 0.000674786 | 0.008271573 | Reproduction |
| S21 | LMA | Toll-like receptor 9 level | 5 | 13 | 0.005817455 | 0.041710052 | Health |
| S21 | LMA | Total shear work | 3 | 5 | 0.007991875 | 0.049785452 | Meat and Carcass |
| S21 | LMD | Abdominal fat weight | 14 | 40 | 1.75012E-05 | 0.000286868 | Meat and Carcass |
| S21 | LMD | Actinobacillus pleuropneumoniae susceptibility | 17 | 78 | 0.001515207 | 0.013932517 | Health |
| S21 | LMD | Adipocyte diameter | 22 | 66 | 2.02324E-07 | 5.08507E-06 | Meat and Carcass |
| S21 | LMD | androstenone, laboratory | 79 | 111 | 8.8249E-54 | 1.109E-51 | Meat and Carcass |
| S21 | LMD | Arachidic acid content | 16 | 39 | 3.65779E-07 | 8.61866E-06 | Meat and Carcass |
| S21 | LMD | Aspartate aminotransferase activity | 14 | 22 | 1.36772E-09 | 5.72921E-08 | Health |
| S21 | LMD | backfat above muscle dorsi | 8 | 26 | 0.002909163 | 0.022849052 | Meat and Carcass |
| S21 | LMD | Backfat at first rib | 12 | 54 | 0.006050578 | 0.041071941 | Meat and Carcass |
| S21 | LMD | Backfat at last rib | 53 | 268 | 9.4712E-07 | 1.98369E-05 | Meat and Carcass |
| S21 | LMD | backfat at mid-back | 15 | 32 | 9.98269E-08 | 3.13623E-06 | Meat and Carcass |
| S21 | LMD | backfat at P2 position | 6 | 12 | 0.000529947 | 0.006054247 | Meat and Carcass |
| S21 | LMD | Backfat weight | 10 | 43 | 0.008451673 | 0.048415539 | Meat and Carcass |
| S21 | LMD | Basophil number | 87 | 102 | 1.81962E-71 | 3.42998E-69 | Health |
| S21 | LMD | Belly weight | 9 | 30 | 0.001963173 | 0.017212002 | Meat and Carcass |
| S21 | LMD | Blood non-esterified fatty acid level | 9 | 11 | 4.38197E-08 | 1.50182E-06 | Health |
| S21 | LMD | body weight (17 weeks) | 4 | 8 | 0.004958707 | 0.03461912 | Production |
| S21 | LMD | Body weight (20 weeks) | 3 | 5 | 0.00847593 | 0.048415539 | Production |
| S21 | LMD | Body weight (birth) | 24 | 135 | 0.00367026 | 0.027131137 | Production |
| S21 | LMD | Body weight (end of test) | 6 | 12 | 0.000529947 | 0.006054247 | Production |
| S21 | LMD | C3c concentration | 11 | 33 | 0.000228678 | 0.00297282 | Health |
| S21 | LMD | Carcass weight (hot) | 21 | 85 | 6.94149E-05 | 0.001046777 | Meat and Carcass |
| S21 | LMD | Conductivity 24 hours post-mortem | 9 | 36 | 0.007479336 | 0.046995164 | Meat and Carcass |
| S21 | LMD | Conductivity 45 minutes post-mortem | 61 | 201 | 7.62718E-16 | 5.75089E-14 | Meat and Carcass |
| S21 | LMD | Creatine kinase level | 6 | 13 | 0.00090126 | 0.009438193 | Health |
| S21 | LMD | Creatinine level | 6 | 15 | 0.002205664 | 0.018076858 | Health |
| S21 | LMD | Diameter of type I muscle fibers | 4 | 9 | 0.008225231 | 0.048415539 | Meat and Carcass |
| S21 | LMD | Ear erectness | 13 | 25 | 1.53089E-07 | 4.12245E-06 | Exterior |
| S21 | LMD | Ear weight | 7 | 21 | 0.003201167 | 0.024629391 | Exterior |
| S21 | LMD | Empty body lipid content | 5 | 13 | 0.006358935 | 0.041333075 | Meat and Carcass |
| S21 | LMD | Enterotoxigenic E. coli susceptibility | 33 | 125 | 1.31095E-07 | 3.80176E-06 | Health |
| S21 | LMD | Estimated carcass lean content | 8 | 30 | 0.00760549 | 0.047004421 | Meat and Carcass |
| S21 | LMD | Fat area percentage in carcass | 13 | 34 | 1.15302E-05 | 0.000206995 | Meat and Carcass |
| S21 | LMD | Feet weight | 4 | 5 | 0.000453481 | 0.00551492 | Meat and Carcass |
| S21 | LMD | Firmness | 17 | 24 | 1.58022E-12 | 9.92907E-11 | Meat and Carcass |
| S21 | LMD | Ham fat thickness | 5 | 12 | 0.004259829 | 0.030301047 | Meat and Carcass |
| S21 | LMD | Ham weight | 22 | 129 | 0.008614352 | 0.048471801 | Meat and Carcass |
| S21 | LMD | Head weight | 204 | 351 | 8.2301E-112 | 3.1028E-109 | Meat and Carcass |
| S21 | LMD | Hemoglobin | 13 | 62 | 0.007312331 | 0.046724555 | Health |
| S21 | LMD | Impedance 24 hours postmortem | 12 | 44 | 0.00094735 | 0.009652727 | Meat and Carcass |
| S21 | LMD | Interleukin-10 level | 8 | 25 | 0.002204428 | 0.018076858 | Health |
| S21 | LMD | Intermuscular fat content | 13 | 51 | 0.001177356 | 0.011680607 | Meat and Carcass |
| S21 | LMD | Lactate dehydrogenase level | 3 | 4 | 0.003662461 | 0.027131137 | Health |
| S21 | LMD | Leaf fat weight | 12 | 37 | 0.000160915 | 0.00224685 | Meat and Carcass |
| S21 | LMD | Lip thickness | 21 | 21 | 8.76378E-22 | 8.25986E-20 | Meat and Carcass |
| S21 | LMD | Loin fat percentage | 5 | 9 | 0.000875552 | 0.009430942 | Meat and Carcass |
| S21 | LMD | Loin muscle area | 82 | 401 | 2.19127E-10 | 1.03264E-08 | Meat and Carcass |
| S21 | LMD | Meat color b* | 24 | 111 | 0.000208604 | 0.002808702 | Meat and Carcass |
| S21 | LMD | Meat color-a | 7 | 20 | 0.002332815 | 0.018712151 | Meat and Carcass |
| S21 | LMD | Mouth width, including lips | 10 | 10 | 9.55162E-11 | 5.14423E-09 | Meat and Carcass |
| S21 | LMD | Muscle moisture percentage | 18 | 87 | 0.002081829 | 0.017837485 | Meat and Carcass |
| S21 | LMD | muscle protein percentage | 9 | 29 | 0.001505949 | 0.013932517 | Meat and Carcass |
| S21 | LMD | NADP-malate dehydrogenase activity | 5 | 10 | 0.001607393 | 0.014428267 | Meat and Carcass |
| S21 | LMD | Nonfunctional nipples | 11 | 40 | 0.001419673 | 0.013723507 | Reproduction |
| S21 | LMD | Osteochondrosis score | 5 | 14 | 0.009089124 | 0.049660865 | Exterior |
| S21 | LMD | pH 24 hr post-mortem (loin) | 45 | 252 | 8.37809E-05 | 0.001214823 | Meat and Carcass |
| S21 | LMD | pH 45 minutes post mortem | 23 | 109 | 0.000405682 | 0.005098073 | Meat and Carcass |
| S21 | LMD | pH for Semimembranosus | 14 | 38 | 8.8354E-06 | 0.000166547 | Meat and Carcass |
| S21 | LMD | Salmonella count in liver and spleen | 5 | 12 | 0.004259829 | 0.030301047 | Health |
| S21 | LMD | Scapula length | 8 | 29 | 0.006100872 | 0.041071941 | Production |
| S21 | LMD | Shear force at first peak | 14 | 31 | 4.75252E-07 | 1.05394E-05 | Meat and Carcass |
| S21 | LMD | Shoulder subcutaneous fat thickness | 21 | 69 | 2.06356E-06 | 4.09453E-05 | Meat and Carcass |
| S21 | LMD | Shoulder weight | 30 | 96 | 7.35768E-09 | 2.77385E-07 | Meat and Carcass |
| S21 | LMD | Side fat thickness | 5 | 14 | 0.009089124 | 0.049660865 | Meat and Carcass |
| S21 | LMD | Skin percentage | 3 | 5 | 0.00847593 | 0.048415539 | Meat and Carcass |
| S21 | LMD | Testicular percentage | 6 | 8 | 2.28802E-05 | 0.00035941 | Reproduction |
| S21 | LMD | Testicular weight | 7 | 17 | 0.000765197 | 0.008484688 | Reproduction |
| S21 | LMD | Toll-like receptor 9 level | 5 | 13 | 0.006358935 | 0.041333075 | Health |
| S21 | LMD | Total shear work | 3 | 5 | 0.00847593 | 0.048415539 | Meat and Carcass |
| S21 | LMD | Water holding capacity | 12 | 30 | 1.45857E-05 | 0.000249946 | Meat and Carcass |
| S21 | LMP | Abdominal fat weight | 11 | 40 | 1.22E-05 | 0.000177192 | Meat and Carcass |
| S21 | LMP | Actinobacillus pleuropneumoniae susceptibility | 15 | 78 | 3.81E-05 | 0.000443974 | Health |
| S21 | LMP | Adipocyte diameter | 12 | 66 | 0.000384086 | 0.003386941 | Meat and Carcass |
| S21 | LMP | Average backfat thickness | 49 | 437 | 9.36E-06 | 0.000151242 | Meat and Carcass |
| S21 | LMP | Average daily gain | 72 | 734 | 1.13E-05 | 0.000172871 | Production |
| S21 | LMP | Backfat at last rib | 28 | 268 | 0.002075479 | 0.015486264 | Meat and Carcass |
| S21 | LMP | backfat at mid-back | 12 | 32 | 1.11E-07 | 4.62E-06 | Meat and Carcass |
| S21 | LMP | backfat at P2 position | 6 | 12 | 2.64E-05 | 0.000348656 | Meat and Carcass |
| S21 | LMP | Backfat weight | 8 | 43 | 0.00303639 | 0.021037844 | Meat and Carcass |
| S21 | LMP | Belly weight | 6 | 30 | 0.006865917 | 0.040945163 | Meat and Carcass |
| S21 | LMP | Body height | 12 | 34 | 2.42E-07 | 7.04E-06 | Production |
| S21 | LMP | body length | 13 | 79 | 0.000609865 | 0.004929739 | Production |
| S21 | LMP | body weight (17 weeks) | 3 | 8 | 0.008850814 | 0.049530518 | Production |
| S21 | LMP | Body weight (20 weeks) | 3 | 5 | 0.001804461 | 0.013818375 | Production |
| S21 | LMP | Body weight (30 weeks) | 5 | 21 | 0.006188598 | 0.038316638 | Production |
| S21 | LMP | Body weight (end of test) | 5 | 12 | 0.000373598 | 0.003386941 | Production |
| S21 | LMP | Cannon bone circumference | 23 | 75 | 2.18E-11 | 1.27E-09 | Exterior |
| S21 | LMP | Carcass length | 25 | 197 | 0.000211594 | 0.001986255 | Meat and Carcass |
| S21 | LMP | Carcass weight (hot) | 18 | 85 | 1.56E-06 | 3.49E-05 | Meat and Carcass |
| S21 | LMP | Conformation score | 7 | 22 | 0.000176281 | 0.001768891 | Exterior |
| S21 | LMP | Creatine kinase level | 5 | 13 | 0.000578014 | 0.004805775 | Health |
| S21 | LMP | Creatinine level | 5 | 15 | 0.001222863 | 0.009617655 | Health |
| S21 | LMP | Ear erectness | 8 | 25 | 5.76E-05 | 0.000621329 | Exterior |
| S21 | LMP | Facial morphology | 8 | 13 | 1.29E-07 | 4.69E-06 | Exterior |
| S21 | LMP | Fat area percentage in carcass | 12 | 34 | 2.42E-07 | 7.04E-06 | Meat and Carcass |
| S21 | LMP | Femur length | 12 | 45 | 6.98E-06 | 0.000120859 | Production |
| S21 | LMP | Firmness | 15 | 24 | 2.25E-13 | 2.18E-11 | Meat and Carcass |
| S21 | LMP | Ham weight | 16 | 129 | 0.003435782 | 0.023251453 | Meat and Carcass |
| S21 | LMP | Head weight | 203 | 351 | 1.75E-157 | 5.09E-155 | Meat and Carcass |
| S21 | LMP | Hemoglobin | 10 | 62 | 0.002934514 | 0.020827894 | Health |
| S21 | LMP | Hip bone length | 7 | 11 | 6.02E-07 | 1.59E-05 | Production |
| S21 | LMP | Humerus length | 12 | 52 | 3.43E-05 | 0.00043343 | Production |
| S21 | LMP | Leaf fat weight | 12 | 37 | 6.94E-07 | 1.68E-05 | Meat and Carcass |
| S21 | LMP | Lip thickness | 21 | 21 | 1.05E-26 | 1.52E-24 | Meat and Carcass |
| S21 | LMP | Liver weight | 8 | 29 | 0.000185656 | 0.001800859 | Meat and Carcass |
| S21 | LMP | Loin muscle area | 36 | 401 | 0.006894546 | 0.040945163 | Meat and Carcass |
| S21 | LMP | Loin muscle depth | 17 | 90 | 1.52E-05 | 0.000210724 | Meat and Carcass |
| S21 | LMP | Mean corpuscular hemoglobin concentration | 31 | 312 | 0.002719849 | 0.019786905 | Health |
| S21 | LMP | Meat color b* | 14 | 111 | 0.005143866 | 0.033263668 | Meat and Carcass |
| S21 | LMP | Mouth width, including lips | 10 | 10 | 4.38E-13 | 3.19E-11 | Meat and Carcass |
| S21 | LMP | Muscle moisture percentage | 12 | 87 | 0.004503067 | 0.029781649 | Meat and Carcass eQTL |
| S21 | LMP | NADP-malate dehydrogenase activity | 5 | 10 | 0.000131165 | 0.001363183 | Meat and Carcass |
| S21 | LMP | Percentage type I fibers | 6 | 19 | 0.000543454 | 0.004651329 | Meat and Carcass |
| S21 | LMP | pH for Semimembranosus | 11 | 38 | 7.06E-06 | 0.000120859 | Meat and Carcass |
| S21 | LMP | Phosphate level | 5 | 22 | 0.007630678 | 0.044410546 | Health |
| S21 | LMP | Potassium level | 3 | 8 | 0.008850814 | 0.049530518 | Health |
| S21 | LMP | Scapula length | 6 | 29 | 0.005772108 | 0.036514856 | Production |
| S21 | LMP | Shear force at first peak | 14 | 31 | 5.09E-10 | 2.47E-08 | Meat and Carcass |
| S21 | LMP | Shoulder subcutaneous fat thickness | 14 | 69 | 3.68E-05 | 0.000443974 | Meat and Carcass |
| S21 | LMP | Tibia length | 12 | 54 | 5.11E-05 | 0.000571931 | Production |
| S21 | LMP | Ulna length | 12 | 45 | 6.98E-06 | 0.000120859 | Production |
| S21 | LMP | Water holding capacity | 10 | 30 | 4.48E-06 | 9.32E-05 | Meat and Carcass |
| S21 | TN | Abdominal fat weight | 7 | 40 | 0.000124292 | 0.003790903 | Meat and Carcass |
| S21 | TN | Average backfat thickness | 27 | 437 | 0.000170076 | 0.004610939 | Meat and Carcass |
| S21 | TN | Average daily gain | 38 | 734 | 0.000356091 | 0.007240515 | Production |
| S21 | TN | backfat above muscle dorsi | 5 | 26 | 0.000751882 | 0.011466196 | Meat and Carcass |
| S21 | TN | Backfat at last rib | 21 | 268 | 3.33E-05 | 0.001626896 | Meat and Carcass |
| S21 | TN | Backfat at tenth rib | 21 | 221 | 1.71E-06 | 0.000174657 | Meat and Carcass |
| S21 | TN | body weight (17 weeks) | 3 | 8 | 0.001169487 | 0.016240972 | Production |
| S21 | TN | Body weight (20 weeks) | 3 | 5 | 0.000222748 | 0.004940961 | Production |
| S21 | TN | Body weight (end of test) | 3 | 12 | 0.004217756 | 0.036754731 | Production |
| S21 | TN | Body weight (weaning) | 5 | 30 | 0.001482275 | 0.018083755 | Production |
| S21 | TN | Carcass length | 17 | 197 | 5.58E-05 | 0.002268961 | Meat and Carcass |
| S21 | TN | Carcass weight (hot) | 8 | 85 | 0.002984464 | 0.029870331 | Meat and Carcass |
| S21 | TN | Creatine kinase level | 3 | 13 | 0.005367507 | 0.042247476 | Health |
| S21 | TN | Creatinine level | 6 | 15 | 2.15E-06 | 0.000174657 | Health |
| S21 | TN | Diameter of type I muscle fibers | 3 | 9 | 0.001717033 | 0.019043461 | Meat and Carcass |
| S21 | TN | Ear erectness | 5 | 25 | 0.000621806 | 0.010114711 | Exterior |
| S21 | TN | Empty body lipid content | 3 | 13 | 0.005367507 | 0.042247476 | Meat and Carcass |
| S21 | TN | External fat on ham | 5 | 25 | 0.000621806 | 0.010114711 | Meat and Carcass |
| S21 | TN | Fat area percentage in carcass | 15 | 34 | 6.85E-15 | 1.67E-12 | Meat and Carcass |
| S21 | TN | Ham weight | 11 | 129 | 0.001216829 | 0.016240972 | Meat and Carcass |
| S21 | TN | Loin fat percentage | 3 | 9 | 0.001717033 | 0.019043461 | Meat and Carcass |
| S21 | TN | Loin muscle area | 28 | 401 | 1.53E-05 | 0.000935308 | Meat and Carcass |
| S21 | TN | Meat color a* | 13 | 188 | 0.003060485 | 0.029870331 | Meat and Carcass |
| S21 | TN | Meat color-a | 5 | 20 | 0.000204254 | 0.004940961 | Meat and Carcass |
| S21 | TN | muscle protein percentage | 5 | 29 | 0.001264666 | 0.016240972 | Meat and Carcass |
| S21 | TN | pH for Semimembranosus | 7 | 38 | 8.84E-05 | 0.003081831 | Meat and Carcass |
| S21 | TN | Phosphate level | 4 | 22 | 0.003212414 | 0.03014727 | Health |
| S21 | TN | Shoulder subcutaneous fat thickness | 7 | 69 | 0.003522894 | 0.031836521 | Meat and Carcass |
| S21 | TN | Smell intensity | 2 | 3 | 0.002400632 | 0.025467575 | Meat and Carcass |
| S21 | TN | subjective boar flavor in lean | 2 | 4 | 0.004710223 | 0.039630844 | Meat and Carcass |
| S21 | TN | White blood cell number | 7 | 50 | 0.000520032 | 0.0097606 | Health |
| S22 | ADG | Abdominal fat weight | 6 | 40 | 0.006498808 | 0.04679142 | Meat and Carcass |
| S22 | ADG | Adipocyte diameter | 12 | 66 | 1.95E-05 | 0.000446195 | Meat and Carcass |
| S22 | ADG | Average backfat thickness | 41 | 437 | 2.16E-06 | 6.79E-05 | Meat and Carcass |
| S22 | ADG | backfat above muscle dorsi | 6 | 26 | 0.000646418 | 0.010866221 | Meat and Carcass |
| S22 | ADG | Backfat at last rib | 24 | 268 | 0.000516795 | 0.010782113 | Meat and Carcass |
| S22 | ADG | backfat at mid-back | 9 | 32 | 5.11E-06 | 0.000143066 | Meat and Carcass |
| S22 | ADG | Backfat weight | 7 | 43 | 0.002083205 | 0.022824678 | Meat and Carcass |
| S22 | ADG | Creatinine level | 4 | 15 | 0.003048831 | 0.029550205 | Health |
| S22 | ADG | Ear erectness | 5 | 25 | 0.003600003 | 0.031785965 | Exterior |
| S22 | ADG | Ear weight | 5 | 21 | 0.001587586 | 0.0210564 | Exterior |
| S22 | ADG | Feet weight | 3 | 5 | 0.000719279 | 0.011328648 | Meat and Carcass |
| S22 | ADG | Femur length | 7 | 45 | 0.002725569 | 0.028618471 | Production |
| S22 | ADG | Head weight | 201 | 351 | 3.71E-183 | 9.34E-181 | Meat and Carcass |
| S22 | ADG | Humerus length | 7 | 52 | 0.0062167 | 0.046076719 | Production |
| S22 | ADG | Leaf fat weight | 7 | 37 | 0.00083053 | 0.01231138 | Meat and Carcass |
| S22 | ADG | Lip thickness | 21 | 21 | 1.35E-29 | 1.71E-27 | Meat and Carcass |
| S22 | ADG | Litter weight, piglets born alive | 5 | 24 | 0.002983136 | 0.029550205 | Reproduction |
| S22 | ADG | Liver weight | 6 | 29 | 0.001196048 | 0.01674467 | Meat and Carcass |
| S22 | ADG | Loin fat percentage | 3 | 9 | 0.005314183 | 0.041849191 | Meat and Carcass |
| S22 | ADG | Loin muscle area | 42 | 401 | 8.52E-08 | 3.58E-06 | Meat and Carcass |
| S22 | ADG | Loin muscle depth | 10 | 90 | 0.00489079 | 0.040826373 | Meat and Carcass |
| S22 | ADG | Mean corpuscular hemoglobin concentration | 54 | 312 | 1.21E-18 | 1.01E-16 | Health |
| S22 | ADG | Mean corpuscular volume | 47 | 498 | 3.36E-07 | 1.21E-05 | Health |
| S22 | ADG | Meat color a* | 22 | 188 | 1.79E-05 | 0.000446195 | Meat and Carcass |
| S22 | ADG | Melanoma susceptibility | 8 | 65 | 0.006152779 | 0.046076719 | Health |
| S22 | ADG | Mouth width, including lips | 10 | 10 | 1.87E-14 | 1.18E-12 | Meat and Carcass |
| S22 | ADG | NADP-malate dehydrogenase activity | 7 | 10 | 2.65E-08 | 1.34E-06 | Meat and Carcass |
| S22 | ADG | NADPH-generating enzyme activity | 4 | 10 | 0.00055622 | 0.010782113 | Meat and Carcass |
| S22 | ADG | Palmitic acid to myristic acid ratio | 5 | 22 | 0.001983242 | 0.022717138 | Meat and Carcass |
| S22 | ADG | pH for Semimembranosus | 6 | 38 | 0.005022292 | 0.040826373 | Meat and Carcass |
| S22 | ADG | Phosphate level | 5 | 22 | 0.001983242 | 0.022717138 | Health |
| S22 | ADG | Potassium level | 3 | 8 | 0.003657909 | 0.031785965 | Health |
| S22 | ADG | Shoulder subcutaneous fat thickness | 10 | 69 | 0.000646799 | 0.010866221 | Meat and Carcass |
| S22 | ADG | Tibia length | 8 | 54 | 0.001909811 | 0.022717138 | Production |
| S22 | ADG | Trimmed wholesale product / live weight | 3 | 8 | 0.003657909 | 0.031785965 | Meat and Carcass |
| S22 | BF | Abdominal fat weight | 8 | 40 | 0.002477236 | 0.017920431 | Meat and Carcass |
| S22 | BF | Actinobacillus pleuropneumoniae susceptibility | 15 | 78 | 6.30E-05 | 0.001259665 | Health |
| S22 | BF | Adipocyte diameter | 16 | 66 | 1.55E-06 | 5.86E-05 | Meat and Carcass |
| S22 | BF | Arachidic acid to stearic acid ratio | 16 | 121 | 0.002767798 | 0.019605236 | Meat and Carcass |
| S22 | BF | Arachidonic acid content | 7 | 25 | 0.000555557 | 0.006296318 | Meat and Carcass |
| S22 | BF | Aspartate aminotransferase activity | 14 | 22 | 1.83E-12 | 6.24E-10 | Health |
| S22 | BF | Average backfat thickness | 49 | 437 | 2.92E-05 | 0.000767342 | Meat and Carcass |
| S22 | BF | Average daily gain | 78 | 734 | 1.25E-06 | 5.42E-05 | Production |
| S22 | BF | Backfat at last rib | 38 | 268 | 1.08E-06 | 5.42E-05 | Meat and Carcass |
| S22 | BF | backfat at mid-back | 9 | 32 | 8.76E-05 | 0.001489585 | Meat and Carcass |
| S22 | BF | backfat at P2 position | 6 | 12 | 3.39E-05 | 0.000767342 | Meat and Carcass |
| S22 | BF | Backfat weight | 11 | 43 | 3.87E-05 | 0.000821554 | Meat and Carcass |
| S22 | BF | Body weight (20 weeks) | 3 | 5 | 0.002050782 | 0.015846955 | Production |
| S22 | BF | Body weight (birth) | 16 | 135 | 0.008075526 | 0.046415341 | Production |
| S22 | BF | Body weight (end of test) | 6 | 12 | 3.39E-05 | 0.000767342 | Production |
| S22 | BF | Body weight (weaning) | 6 | 30 | 0.008463974 | 0.046415341 | Production |
| S22 | BF | C3c concentration | 10 | 33 | 1.73E-05 | 0.000533781 | Health |
| S22 | BF | Carcass length | 23 | 197 | 0.002099541 | 0.015863202 | Meat and Carcass |
| S22 | BF | Carcass weight (hot) | 19 | 85 | 6.47E-07 | 4.40E-05 | Meat and Carcass |
| S22 | BF | CD4-positive, CD8-positive leukocyte percentage | 5 | 18 | 0.003648247 | 0.024321649 | Health |
| S22 | BF | Conductivity 24 hours post-mortem | 8 | 36 | 0.001210059 | 0.010549232 | Meat and Carcass |
| S22 | BF | Creatine kinase level | 5 | 13 | 0.00070744 | 0.007516545 | Health |
| S22 | BF | Creatinine level | 6 | 15 | 0.000156433 | 0.002127489 | Health |
| S22 | BF | Daily feed intake | 14 | 103 | 0.003842832 | 0.025120365 | Production |
| S22 | BF | Ear erectness | 8 | 25 | 7.87E-05 | 0.001486717 | Exterior |
| S22 | BF | Ear weight | 7 | 21 | 0.000166599 | 0.002178608 | Exterior |
| S22 | BF | Estimated carcass lean content | 6 | 30 | 0.008463974 | 0.046415341 | Meat and Carcass |
| S22 | BF | External fat on ham | 7 | 25 | 0.000555557 | 0.006296318 | Meat and Carcass |
| S22 | BF | Fat androstenone level | 28 | 169 | 1.28E-06 | 5.42E-05 | Meat and Carcass |
| S22 | BF | Fat area percentage in carcass | 12 | 34 | 3.88E-07 | 3.30E-05 | Meat and Carcass |
| S22 | BF | Ham fat thickness | 5 | 12 | 0.000458279 | 0.005564822 | Meat and Carcass |
| S22 | BF | Ham weight | 20 | 129 | 0.000104685 | 0.001617857 | Meat and Carcass |
| S22 | BF | Interferon-gamma level | 5 | 21 | 0.007441695 | 0.043623727 | Health |
| S22 | BF | Interferon-gamma to interleukin-10 ratio | 4 | 9 | 0.001345 | 0.011432501 | Health |
| S22 | BF | Interleukin-2 level | 3 | 4 | 0.000859355 | 0.007923594 | Health |
| S22 | BF | Lactate dehydrogenase level | 3 | 4 | 0.000859355 | 0.007923594 | Health |
| S22 | BF | Linoleic acid content | 13 | 79 | 0.000917266 | 0.008207113 | Meat and Carcass |
| S22 | BF | Loin and neck meat weight | 6 | 27 | 0.004930428 | 0.029934743 | Meat and Carcass |
| S22 | BF | Loin fat percentage | 5 | 9 | 8.51E-05 | 0.001489585 | Meat and Carcass |
| S22 | BF | Loin muscle area | 44 | 401 | 0.000119802 | 0.00169719 | Meat and Carcass |
| S22 | BF | Loin muscle depth | 12 | 90 | 0.008308007 | 0.046415341 | Meat and Carcass |
| S22 | BF | Meat color a* | 35 | 188 | 2.89E-09 | 4.92E-07 | Meat and Carcass |
| S22 | BF | Meat color chroma | 3 | 6 | 0.003915822 | 0.025120365 | Meat and Carcass |
| S22 | BF | Meat color-a | 7 | 20 | 0.000117212 | 0.00169719 | Meat and Carcass |
| S22 | BF | Muscle moisture percentage | 12 | 87 | 0.00635378 | 0.037899743 | Meat and Carcass |
| S22 | BF | Nonfunctional nipples | 8 | 40 | 0.002477236 | 0.017920431 | Reproduction |
| S22 | BF | Number of litters | 5 | 19 | 0.004705922 | 0.029091154 | Reproduction |
| S22 | BF | Number weaned | 11 | 42 | 3.04E-05 | 0.000767342 | Reproduction |
| S22 | BF | Palmitic acid to myristic acid ratio | 10 | 22 | 2.24E-07 | 2.54E-05 | Meat and Carcass |
| S22 | BF | Percentage type I fibers | 5 | 19 | 0.004705922 | 0.029091154 | Meat and Carcass |
| S22 | BF | Percentage type IIb fibers | 7 | 30 | 0.00180097 | 0.014579285 | Meat and Carcass |
| S22 | BF | pH 24 hr post-mortem (loin) | 34 | 252 | 1.15E-05 | 0.000391791 | Meat and Carcass |
| S22 | BF | PH for Longissmus dorsi | 17 | 134 | 0.003229228 | 0.02195875 | Meat and Carcass |
| S22 | BF | pH for Semimembranosus | 9 | 38 | 0.000365772 | 0.004606023 | Meat and Carcass |
| S22 | BF | Physis score | 4 | 8 | 0.00078504 | 0.007923594 | Production |
| S22 | BF | Shoulder meat weight | 6 | 22 | 0.001613426 | 0.013379631 | Meat and Carcass |
| S22 | BF | Shoulder subcutaneous fat thickness | 12 | 69 | 0.000862273 | 0.007923594 | Meat and Carcass |
| S22 | BF | Side fat thickness | 6 | 14 | 9.90E-05 | 0.001602185 | Meat and Carcass |
| S22 | BF | Stiffness | 3 | 5 | 0.002050782 | 0.015846955 | Meat and Carcass |
| S22 | BF | subjective boar flavor in lean | 3 | 4 | 0.000859355 | 0.007923594 | Meat and Carcass |
| S22 | BF | Toll-like receptor 9 level | 5 | 13 | 0.00070744 | 0.007516545 | Health |
| S22 | BF | White blood cell number | 9 | 50 | 0.002913526 | 0.020216306 | Health |
| S22 | BL | Bilirubin level | 1 | 5 | 0.010671357 | 0.044510277 | Health |
| S22 | BL | CD4-negative, CD8-positive leukocyte percentage | 1 | 5 | 0.010671357 | 0.044510277 | Health |
| S22 | BL | CD4-positive leukocyte number | 1 | 2 | 0.004282068 | 0.027298184 | Health |
| S22 | BL | conA-induced cell proliferation | 1 | 2 | 0.004282068 | 0.027298184 | Health |
| S22 | BL | Days to 100 kg | 4 | 146 | 0.000277099 | 0.014132069 | Production |
| S22 | BL | Ear area | 2 | 75 | 0.011345757 | 0.044510277 | Exterior |
| S22 | BL | Feet weight | 1 | 5 | 0.010671357 | 0.044510277 | Meat and Carcass |
| S22 | BL | MHCII-positive leukocyte number | 1 | 1 | 0.002143295 | 0.027298184 | Health |
| S22 | BL | Number of stillborn | 3 | 130 | 0.002765797 | 0.027298184 | Reproduction |
| S22 | BL | Number of visits to feeder per day | 2 | 35 | 0.002570104 | 0.027298184 | Production |
| S22 | BL | PWM-induced cell proliferation | 1 | 2 | 0.004282068 | 0.027298184 | Health |
| S22 | BL | Temperature 40 minutes post mortem (loin) | 1 | 3 | 0.00641633 | 0.036359204 | Meat and Carcass |
| S22 | BL | Uterine capacity | 1 | 2 | 0.004282068 | 0.027298184 | Reproduction |
| S22 | LMA | backfat at mid-back | 5 | 32 | 0.002632307 | 0.031094128 | Meat and Carcass |
| S22 | LMA | Basophil number | 86 | 102 | 7.82E-115 | 7.39E-113 | Health |
| S22 | LMA | Blood non-esterified fatty acid level | 9 | 11 | 1.13E-12 | 3.55E-11 | Health |
| S22 | LMA | Feet weight | 3 | 5 | 0.000268718 | 0.004232304 | Meat and Carcass |
| S22 | LMA | Femur length | 6 | 45 | 0.002317653 | 0.029202424 | Production |
| S22 | LMA | Head weight | 198 | 351 | 1.67E-209 | 3.15E-207 | Meat and Carcass |
| S22 | LMA | Hemolytic complement activity (alternative pathway) | 3 | 11 | 0.00386548 | 0.040587544 | Health |
| S22 | LMA | Humerus length | 6 | 52 | 0.004842024 | 0.047521802 | Production |
| S22 | LMA | Leaf fat weight | 5 | 37 | 0.005028762 | 0.047521802 | Meat and Carcass |
| S22 | LMA | Lip thickness | 21 | 21 | 1.14E-32 | 7.15E-31 | Meat and Carcass |
| S22 | LMA | Loin muscle area | 38 | 401 | 7.89E-10 | 1.86E-08 | Meat and Carcass |
| S22 | LMA | Loin muscle depth | 11 | 90 | 9.12E-05 | 0.001723834 | Meat and Carcass |
| S22 | LMA | Mean corpuscular hemoglobin concentration | 48 | 312 | 2.20E-20 | 1.04E-18 | Health |
| S22 | LMA | Mean corpuscular volume | 45 | 498 | 1.04E-10 | 2.82E-09 | Health |
| S22 | LMA | Mouth width, including lips | 10 | 10 | 6.52E-16 | 2.47E-14 | Meat and Carcass |
| S22 | LMA | NADP-malate dehydrogenase activity | 5 | 10 | 5.74E-06 | 0.000120589 | Meat and Carcass |
| S22 | LMA | NADPH-generating enzyme activity | 3 | 10 | 0.002876012 | 0.031974487 | Meat and Carcass |
| S22 | LMA | Percentage type I fibers | 4 | 19 | 0.002296368 | 0.029202424 | Meat and Carcass |
| S22 | LMA | Percentage type IIb fibers | 6 | 30 | 0.000248802 | 0.004232304 | Meat and Carcass |
| S22 | LMA | Tibia length | 7 | 54 | 0.00120736 | 0.017553162 | Production |
| S22 | LMD | Abdominal fat weight | 8 | 40 | 3.02527E-05 | 0.000547791 | Meat and Carcass |
| S22 | LMD | Actinobacillus pleuropneumoniae susceptibility | 12 | 78 | 5.90772E-06 | 0.000162554 | Health |
| S22 | LMD | Adipocyte diameter | 14 | 66 | 1.51265E-08 | 1.78493E-06 | Meat and Carcass |
| S22 | LMD | Average backfat thickness | 46 | 437 | 1.18875E-12 | 2.80544E-10 | Meat and Carcass |
| S22 | LMD | Average daily gain | 39 | 734 | 0.001225637 | 0.011125009 | Production |
| S22 | LMD | Backfat at first rib | 9 | 54 | 4.48879E-05 | 0.000706236 | Meat and Carcass |
| S22 | LMD | Backfat at last rib | 23 | 268 | 1.62847E-05 | 0.000349381 | Meat and Carcass |
| S22 | LMD | backfat at mid-back | 8 | 32 | 5.17561E-06 | 0.000162554 | Meat and Carcass |
| S22 | LMD | Backfat thickness between 3rd and 4th rib | 5 | 34 | 0.004055167 | 0.02586539 | Meat and Carcass |
| S22 | LMD | Backfat weight | 8 | 43 | 5.24603E-05 | 0.00077379 | Meat and Carcass |
| S22 | LMD | Belly weight | 5 | 30 | 0.002304271 | 0.016994 | Meat and Carcass |
| S22 | LMD | Body weight (150 days) | 5 | 29 | 0.001970885 | 0.016038925 | Production |
| S22 | LMD | body weight (17 weeks) | 4 | 8 | 6.2687E-05 | 0.000870243 | Production |
| S22 | LMD | Body weight (end of test) | 6 | 12 | 7.69836E-07 | 3.63363E-05 | Production |
| S22 | LMD | Body weight (slaughter) | 8 | 46 | 8.68233E-05 | 0.00113835 | Production |
| S22 | LMD | C3c concentration | 8 | 33 | 6.64306E-06 | 0.000162554 | Health |
| S22 | LMD | Carcass length | 16 | 197 | 0.000559154 | 0.00549835 | Meat and Carcass |
| S22 | LMD | Carcass weight (hot) | 13 | 85 | 2.60475E-06 | 0.000102454 | Meat and Carcass |
| S22 | LMD | cis-9-Heptadecenoic acid content | 3 | 5 | 0.000300025 | 0.00337171 | Meat and Carcass |
| S22 | LMD | Conductivity 24 hours post-mortem | 7 | 36 | 0.000115327 | 0.001432481 | Meat and Carcass |
| S22 | LMD | Conductivity 45 minutes post-mortem | 23 | 201 | 1.14288E-07 | 8.99068E-06 | Meat and Carcass |
| S22 | LMD | Cooking loss | 8 | 80 | 0.003765743 | 0.025313544 | Meat and Carcass |
| S22 | LMD | Creatine kinase level | 5 | 13 | 3.24961E-05 | 0.000547791 | Health |
| S22 | LMD | Dressing percentage | 18 | 288 | 0.004952581 | 0.030758138 | Meat and Carcass |
| S22 | LMD | Ear weight | 4 | 21 | 0.003861388 | 0.025313544 | Exterior |
| S22 | LMD | Empty body protein content | 4 | 12 | 0.000400541 | 0.004296711 | Meat and Carcass |
| S22 | LMD | Estimated carcass lean content | 5 | 30 | 0.002304271 | 0.016994 | Meat and Carcass |
| S22 | LMD | Fat to meat ratio | 4 | 17 | 0.001697646 | 0.014308734 | Meat and Carcass |
| S22 | LMD | Fat-cuts percentage | 7 | 44 | 0.000425141 | 0.004362319 | Meat and Carcass |
| S22 | LMD | Ham weight | 13 | 129 | 0.000232168 | 0.002739582 | Meat and Carcass |
| S22 | LMD | Impedance 24 hours postmortem | 6 | 44 | 0.002483451 | 0.01776044 | Meat and Carcass |
| S22 | LMD | Loin fat percentage | 3 | 9 | 0.002291555 | 0.016994 | Meat and Carcass |
| S22 | LMD | Meat color-a | 4 | 20 | 0.003205175 | 0.022247683 | Meat and Carcass |
| S22 | LMD | NADP-malate dehydrogenase activity | 5 | 10 | 6.88789E-06 | 0.000162554 | Meat and Carcass |
| S22 | LMD | pH 45 minutes post mortem | 11 | 109 | 0.000679321 | 0.006412792 | Meat and Carcass |
| S22 | LMD | pH for Semimembranosus | 5 | 38 | 0.006595431 | 0.039910811 | Meat and Carcass |
| S22 | LMD | Physis score | 3 | 8 | 0.001564373 | 0.013673775 | Production |
| S22 | LMD | Shoulder external fat weight | 7 | 28 | 2.0388E-05 | 0.000400964 | Meat and Carcass |
| S22 | LMD | Shoulder subcutaneous fat thickness | 13 | 69 | 2.17113E-07 | 1.28097E-05 | Meat and Carcass |
| S22 | LMP | Abdominal fat weight | 8 | 40 | 0.001831748 | 0.016180441 | Meat and Carcass |
| S22 | LMP | Actinobacillus pleuropneumoniae susceptibility | 14 | 78 | 0.000142885 | 0.00239144 | Health |
| S22 | LMP | Adipocyte diameter | 16 | 66 | 8.25E-07 | 3.75E-05 | Meat and Carcass |
| S22 | LMP | Arachidic acid to stearic acid ratio | 16 | 121 | 0.001708357 | 0.016180441 | Meat and Carcass |
| S22 | LMP | Arachidonic acid content | 7 | 25 | 0.000415485 | 0.005744535 | Meat and Carcass |
| S22 | LMP | Aspartate aminotransferase activity | 20 | 22 | 3.45E-23 | 5.49E-21 | Health |
| S22 | LMP | Average backfat thickness | 40 | 437 | 0.00307428 | 0.02572687 | Meat and Carcass |
| S22 | LMP | Average daily gain | 72 | 734 | 9.84E-06 | 0.000312966 | Production |
| S22 | LMP | Backfat at last rib | 32 | 268 | 9.06E-05 | 0.001799919 | Meat and Carcass |
| S22 | LMP | backfat at mid-back | 8 | 32 | 0.000378587 | 0.005732895 | Meat and Carcass |
| S22 | LMP | backfat at P2 position | 5 | 12 | 0.000366543 | 0.005732895 | Meat and Carcass |
| S22 | LMP | Backfat weight | 9 | 43 | 0.000681981 | 0.008032219 | Meat and Carcass |
| S22 | LMP | Basophil number | 98 | 102 | 1.54E-116 | 4.89E-114 | Health |
| S22 | LMP | Blood non-esterified fatty acid level | 9 | 11 | 3.60E-10 | 3.82E-08 | Health |
| S22 | LMP | body weight (17 weeks) | 3 | 8 | 0.00875069 | 0.047977922 | Production |
| S22 | LMP | Body weight (20 weeks) | 3 | 5 | 0.001783069 | 0.016180441 | Production |
| S22 | LMP | Body weight (end of test) | 6 | 12 | 2.58E-05 | 0.000619878 | Production |
| S22 | LMP | Body weight (weaning) | 6 | 30 | 0.006732573 | 0.042819164 | Production |
| S22 | LMP | C3c concentration | 10 | 33 | 1.13E-05 | 0.000328115 | Health |
| S22 | LMP | Carcass weight (hot) | 18 | 85 | 1.47E-06 | 5.85E-05 | Meat and Carcass |
| S22 | LMP | Conductivity 24 hours post-mortem | 8 | 36 | 0.000885698 | 0.009712132 | Meat and Carcass |
| S22 | LMP | Creatine kinase level | 4 | 13 | 0.005284201 | 0.035752681 | Health |
| S22 | LMP | Creatinine level | 6 | 15 | 0.000119901 | 0.002178784 | Health |
| S22 | LMP | Daily feed intake | 14 | 103 | 0.002493863 | 0.021433745 | Production |
| S22 | LMP | Diameter of type IIb muscle fibers | 3 | 8 | 0.00875069 | 0.047977922 | Meat and Carcass |
| S22 | LMP | Ear erectness | 8 | 25 | 5.60E-05 | 0.001187113 | Exterior |
| S22 | LMP | Ear weight | 7 | 21 | 0.000123327 | 0.002178784 | Exterior |
| S22 | LMP | Estimated carcass lean content | 6 | 30 | 0.006732573 | 0.042819164 | Meat and Carcass |
| S22 | LMP | External fat on ham | 7 | 25 | 0.000415485 | 0.005744535 | Meat and Carcass |
| S22 | LMP | Fat androstenone level | 28 | 169 | 4.94E-07 | 2.62E-05 | Meat and Carcass |
| S22 | LMP | Fat area percentage in carcass | 11 | 34 | 2.00E-06 | 7.07E-05 | Meat and Carcass |
| S22 | LMP | Ham fat thickness | 4 | 12 | 0.003832821 | 0.027513361 | Meat and Carcass |
| S22 | LMP | Ham weight | 18 | 129 | 0.000476666 | 0.00631583 | Meat and Carcass |
| S22 | LMP | Interleukin-2 level | 3 | 4 | 0.000745509 | 0.008466852 | Health |
| S22 | LMP | Loin and neck meat weight | 6 | 27 | 0.0038934 | 0.027513361 | Meat and Carcass |
| S22 | LMP | Loin fat percentage | 4 | 9 | 0.001122724 | 0.011900879 | Meat and Carcass |
| S22 | LMP | Loin muscle area | 37 | 401 | 0.00376438 | 0.027513361 | Meat and Carcass |
| S22 | LMP | Loin muscle depth | 12 | 90 | 0.005743856 | 0.038053047 | Meat and Carcass |
| S22 | LMP | Meat color a* | 32 | 188 | 4.05E-08 | 3.22E-06 | Meat and Carcass |
| S22 | LMP | Meat color chroma | 3 | 6 | 0.003412188 | 0.027513361 | Meat and Carcass |
| S22 | LMP | Meat color-a | 5 | 20 | 0.004863547 | 0.033621913 | Meat and Carcass |
| S22 | LMP | Myristic acid content | 10 | 70 | 0.006924087 | 0.043173717 | Meat and Carcass |
| S22 | LMP | Number of litters | 5 | 19 | 0.003828281 | 0.027513361 | Reproduction |
| S22 | LMP | Number weaned | 11 | 42 | 1.95E-05 | 0.000516453 | Reproduction |
| S22 | LMP | Palmitic acid to myristic acid ratio | 10 | 22 | 1.43E-07 | 9.08E-06 | Meat and Carcass |
| S22 | LMP | Percentage type I fibers | 5 | 19 | 0.003828281 | 0.027513361 | Meat and Carcass |
| S22 | LMP | Percentage type IIb fibers | 7 | 30 | 0.001364033 | 0.01355508 | Meat and Carcass |
| S22 | LMP | pH 24 hr post-mortem (loin) | 32 | 252 | 2.73E-05 | 0.000619878 | Meat and Carcass |
| S22 | LMP | pH for Semimembranosus | 8 | 38 | 0.001291108 | 0.013244273 | Meat and Carcass |
| S22 | LMP | Physis score | 3 | 8 | 0.00875069 | 0.047977922 | Production |
| S22 | LMP | Potassium level | 3 | 8 | 0.00875069 | 0.047977922 | Health |
| S22 | LMP | Salmonella count in liver and spleen | 4 | 12 | 0.003832821 | 0.027513361 | Health |
| S22 | LMP | Shoulder meat weight | 5 | 22 | 0.007501628 | 0.045009771 | Meat and Carcass |
| S22 | LMP | Shoulder subcutaneous fat thickness | 12 | 69 | 0.000564484 | 0.006937479 | Meat and Carcass |
| S22 | LMP | Side fat thickness | 4 | 14 | 0.007061761 | 0.043185382 | Meat and Carcass |
| S22 | LMP | Stiffness | 3 | 5 | 0.001783069 | 0.016180441 | Meat and Carcass |
| S22 | LMP | Toll-like receptor 9 level | 5 | 13 | 0.000567215 | 0.006937479 | Health |
| S22 | LMP | White blood cell number | 8 | 50 | 0.007664347 | 0.045134488 | Health |
| S22 | TN | Adipocyte diameter | 6 | 66 | 1.08434E-06 | 9.54222E-05 | Meat and Carcass |
| S22 | TN | backfat above muscle dorsi | 3 | 26 | 0.00030466 | 0.008936682 | Meat and Carcass |
| S22 | TN | Carcass length | 5 | 197 | 0.003374581 | 0.049493855 | Meat and Carcass |
| S22 | TN | Carcass weight (hot) | 4 | 85 | 0.000933725 | 0.020541944 | Meat and Carcass |
| S22 | TN | Empty body lipid content | 2 | 13 | 0.001919183 | 0.033777619 | Meat and Carcass |
| S22 | TN | Number of ribs | 3 | 18 | 9.85023E-05 | 0.004334099 | Meat and Carcass |
